# Comparative clinical outcomes and safety of finerenone, SGLT2 inhibitors, RAS inhibitors and ARNI in heart failure with preserved or mildly reduced ejection fraction: a systematic review and network meta-analysis

**DOI:** 10.3389/fphar.2026.1813205

**Published:** 2026-05-15

**Authors:** Zimin Fu, Xin Chen, Haifeng Liu, Jia Yang, Tonghui Jin, Tiejun Liu, Guang Ta

**Affiliations:** 1 College of Chinese Medicine, Changchun University of Chinese Medicine, Changchun, China; 2 Department of Gastroenterology, First Affiliated Hospital to Changchun University of Chinese Medicine, Changchun, China; 3 Department of Rehabilitation, First Affiliated Hospital of Changchun University of Chinese Medicine, Changchun, China

**Keywords:** ARNI, finerenone, heart failure, network meta-analysis, SGLT2i

## Abstract

**Background:**

Although pharmacological therapies for heart failure have advanced, direct comparative evidence on the clinical outcomes and safety of finerenone versus other agents remains limited in HFpEF or HFmrEF.

**Objective:**

A network meta-analysis was conducted to compare clinical outcomes and safety among finerenone, sodium–glucose cotransporter 2 inhibitors (SGLT2i), renin–angiotensin system inhibitors (RASi) and angiotensin receptor–neprilysin inhibitors (ARNI) in patients with HFpEF or HFmrEF.

**Methods:**

A comprehensive systematic search was conducted across PubMed, Embase, the Cochrane Library, and Web of Science from database inception to 3 January 2026. Randomized controlled trials (RCTs) were included, and a network meta-analysis was performed to evaluate cardiovascular (CV) death, worsening heart failure (HF) events, composite renal outcomes, all-cause mortality, total HF hospitalizations, and adverse events.

**Results:**

A total of 27 RCTs involving 65,929 patients were included. Compared with placebo, finerenone was associated with lower risk of CV death (OR = 0.89, 95% CI 0.82–0.95) and worsening HF events (OR = 0.75, 95% CI 0.71–0.79), but higher risk of composite renal outcomes (OR = 1.42, 95% CI 1.10–1.84). No significant differences in CV death or all-cause mortality. In indirect comparisons, finerenone was associated with lower risk of worsening HF events versus canagliflozin (OR = 2.12, 95% CI 1.13–3.98) and RASi (OR = 1.21, 95% CI 1.03–1.42). Sotagliflozin (OR = 0.61, 95% CI 0.50–0.74) and empagliflozin (OR = 0.83, 95% CI 0.69–0.99) were associated with lower risk of total HF hospitalizations. Relative to finerenone, canagliflozin (OR = 1.53, 95% CI 1.25–1.88) and RASi (OR = 1.31, 95% CI 1.10–1.57) were associated with higher risk of adverse events.

**Conclusion:**

Compared with placebo, finerenone was associated with lower risk of CV death and worsening HF events. As most comparisons between interventions were indirect, sotagliflozin and empagliflozin were associated with lower risk of total HF hospitalizations relative to finerenone, whereas ARNI and empagliflozin were associated with lower risk of composite renal outcomes, and canagliflozin and RASi with higher risk of adverse events. No significant differences were observed between interventions for CV death or all-cause mortality.

## 1 Introduction

The prevalence of HF continues to rise, with approximately 64 million individuals affected worldwide (Shahim et al., 2023). Epidemiological data indicate that HF affects approximately 2.5% of the population in the United States (Konstam et al., 2022), whereas corresponding prevalence estimates in South Korea, Thailand, and Australia are around 0.6%, 0.4%, and 1%–2%, respectively (Reyes et al., 2016). HFpEF and HFmrEF represent the predominant HF phenotypes, together accounting for nearly half of all cases (Rosano et al., 2024). Projections further suggest that the global burden of HF will continue to increase through 2050 (Yan et al., 2025). Patients with HF face a substantial risk of CV events (Lee et al., 2023), recurrent hospitalizations, and chronic kidney disease, a burden further amplified by population ageing and the increasing prevalence of CV risk factors (Heidenreich et al., 2013). Despite continued advances in therapeutic strategies, patients with HFpEF/HFmrEF remain exposed to considerable residual CV and renal risk (Patel et al., 2023). Therefore, clarifying the clinical outcomes and safety of different pharmacological therapies in patients with HFpEF or HFmrEF is of important clinical relevance.

Finerenone is a selective, non-steroidal mineralocorticoid receptor antagonist (MRA) that exerts cardioprotective and renoprotective effects by attenuating aberrant activation of the mineralocorticoid receptor (Georgianos and Agarwal, 2022). Prior studies have demonstrated that, among patients with concomitant type 2 diabetes and chronic kidney disease, finerenone reduces the risk of composite CV outcomes comprising CV death, nonfatal myocardial infarction, nonfatal stroke, and HF hospitalization (Bakris et al., 2020; Pitt et al., 2021). The FINEARTS-HF trial further demonstrated that finerenone lowered the risk of a composite endpoint consisting of worsening HF events and CV death in patients with HFmrEF/HFpEF (Solomon et al., 2024). Collectively, the available evidence suggests that finerenone may confer potential CV benefits in patients with HFmrEF/HFpEF.

Previous studies have shown that SGLT2i are associated with improved CV outcomes and a reduced risk of HF hospitalization in patients with HFpEF or HFmrEF, whereas evidence supporting the efficacy of ARNI in the overall HFpEF population remains limited (Xiang et al., 2022). According to the ACC/AHA/HFSA guidelines, SGLT2i are recommended for use in patients with HFmrEF or HFpEF to reduce the risk of HF hospitalization, whereas RASi and ARNI may be considered in this population, although the supporting evidence remains relatively limited (Heidenreich et al., 2022). However, prior studies have primarily focused on outcomes such as CV death, HF hospitalization and all-cause mortality, with comparatively limited systematic evaluation of composite renal outcomes and adverse events, and direct comparisons between different pharmacological therapies remain insufficiently defined (Ji et al., 2023; Zafeiropoulos et al., 2024).

Patients with HFpEF and HFmrEF differ in their clinical characteristics and comorbidity profiles, and both may involve mechanisms such as inflammation and myocardial remodelling (Li et al., 2021). These populations are frequently accompanied by comorbidities, including chronic kidney disease and diabetes mellitus (Patel et al., 2023), thereby underscoring the clinical importance of comparing the safety profiles of different pharmacological therapies in treatment decision-making. Finerenone is primarily associated with hyperkalaemia and changes in renal function (Yu et al., 2025), SGLT2i with volume depletion and an increased risk of infections (Wu et al., 2025), RASi with hypotension and renal-related adverse events (Hernandez et al., 2023), and ARNI predominantly with hypotension-related adverse events (Abdin et al., 2022). Network meta-analysis enables the integration of direct and indirect evidence, thereby allowing systematic comparisons across multiple interventions when head-to-head comparisons are limited. In this context, the present study aimed to systematically compare the clinical outcomes and safety of these therapies in patients with HFpEF or HFmrEF using a network meta-analysis, with the aim of providing more comprehensive comparative evidence.

## 2 Methods

### 2.1 Protocol registration

This systematic review and network meta-analysis was conducted in accordance with the Preferred Reporting Items for Systematic Reviews and Meta-Analyses (PRISMA) statement and its extension for network meta-analyses (PRISMA-NMA) (Hutton et al., 2015), with the PRISMA 2020 checklist provided in Supplementary Table S1. The study protocol was finalized prior to study initiation and was prospectively registered in the International Prospective Register of Systematic Reviews (PROSPERO: CRD420261305793). As this study exclusively synthesized and analyzed aggregated data from previously published literature, without the involvement of individual-level patient information, approval from an ethics committee and informed consent from participants were not required.

### 2.2 Literature search strategy

The databases PubMed, Embase, the Cochrane Library, and Web of Science were systematically searched from their inception to 3 January 2026, with the detailed search strategy provided in Supplementary Table S2. The search strategy was developed in accordance with the PICOS framework and implemented using combinations of Boolean operators (“AND” and “OR”).

### 2.3 Eligibility criteria

Studies were eligible for inclusion if they met all of the following criteria (Abdin et al., 2022): an RCT design (Abraham et al., 2021); enrollment of patients with HF and a left ventricular ejection fraction (LVEF) ≥40%, consistent with the clinical definitions of HFpEF or HFmrEF (Agarwal et al., 2021); evaluation of at least one pharmacological intervention, including finerenone, SGLT2i, RASi, or ARNI; and (Anker et al., 2021) reporting of at least one of the following outcomes: CV death, worsening HF events, a composite renal outcome, all-cause mortality, total HF hospitalizations, or adverse events.

Studies were excluded if they met any of the following criteria (Abdin et al., 2022): non-randomized controlled trials, including conference abstracts, reviews, editorials, observational studies, or animal experiments (Abraham et al., 2021); enrollment of populations predominantly comprising patients with acute heart failure or myocardial infarction (Agarwal et al., 2021); failure to clearly report primary or secondary outcomes, or inability to obtain outcome data.

### 2.4 Data extraction

Study selection and data extraction were performed independently by two investigators (Z.F. and H.L.). An initial screening of titles and abstracts was conducted, followed by full-text assessment of studies deemed potentially eligible. Any discrepancies were resolved through discussion and consensus, with adjudication by a third investigator (T.L.) when necessary. Key study characteristics, including authorship, year of publication, study design, sample size, and interventions, were extracted using a standardized data extraction form.

### 2.5 Outcome definitions and handling

The included outcomes were categorized and organized for analysis. CV death and all-cause mortality were extracted directly as reported in the original studies; worsening HF events included outcomes related to HF exacerbation or clinical deterioration as defined in each study; total HF hospitalizations comprised total or first hospitalization events; composite renal outcomes included significant deterioration in renal function or renal-related adverse events, mainly comprising declines in estimated glomerular filtration rate (eGFR), end-stage renal disease, or renal death; and adverse events were defined as overall adverse events as reported in each study. All outcomes were incorporated into the analysis based on the original study definitions. Given that some studies reported only event counts or incidence proportions without providing hazard ratios (HRs) or repeated-event measures, these outcomes were treated as binary variables and pooled using odds ratios (ORs).

### 2.6 Risk of bias assessment

The risk of bias of the included RCTs was assessed using the Cochrane Risk of Bias tool, version 2 (RoB 2) (Sterne et al., 2019). Assessments were conducted independently by two investigators across five domains: the randomization process, deviations from intended interventions, missing outcome data, measurement of outcomes, and selection of the reported results. For each domain, the risk of bias was judged as low risk, some concerns, or high risk in accordance with the tool’s criteria. Any disagreements arising during the assessment process were resolved through discussion, with arbitration by a third investigator when consensus could not be reached.

### 2.7 Statistical analysis

Statistical analyses were performed using Stata version 18.0 MP. Network evidence plots were constructed for each outcome to illustrate the comparative relationships among interventions and the distribution of sample sizes. Outcomes were treated as binary variables and expressed as ORs with corresponding 95% confidence intervals (CIs). Given that direct comparisons between interventions were relatively limited among the included studies, the majority of comparisons were based on indirect evidence, and in the absence of closed loops within the network, analyses were conducted under a consistency model framework. To minimize the potential impact of between-study differences on indirect comparisons, key potential effect modifiers were extracted and summarized across trials, including mean age, sex distribution, the presence of diabetes and chronic kidney disease, follow-up duration, and background therapies, and were presented in a baseline characteristics table. On this basis, a multivariable random-effects model was applied to compare differences across interventions, and sensitivity analyses were performed to assess the robustness of the main findings. The results were presented as forest plots and league tables, and the surface under the cumulative ranking curve (SUCRA) was calculated to estimate the relative ranking probabilities of each intervention. Funnel plots were used to explore potential publication bias.

## 3 Results

### 3.1 Study selection and characteristics

**FIGURE 1:**
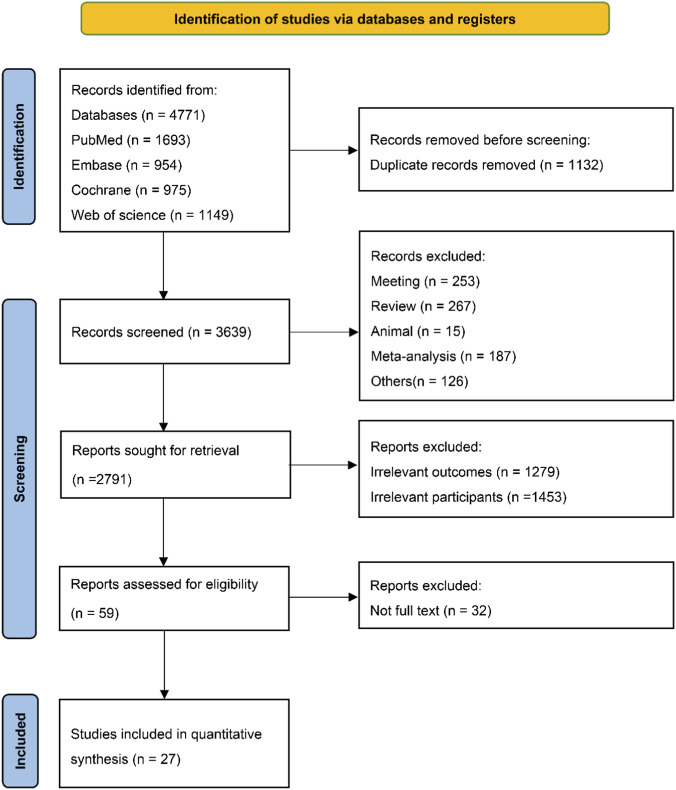
PRISMA flow diagram.

A total of 4,771 records were initially identified, of these, 27 studies were ultimately included in the analysis, comprising 65,929 participants (Figure 1). The baseline characteristics of the included studies are summarized in Table 1. Overall, the mean age of participants was 71.00 years, 55.14% of whom were male. Most studies reported diabetes status, whereas chronic kidney disease status was reported in only a subset of studies. With respect to background therapy, patients generally received standard HF treatments, including ACEi/ARB/ARNI and diuretics, with some studies also reporting concomitant use of beta-blockers, MRAs, and lipid-lowering, anticoagulant, or glucose-lowering therapies (Supplementary Table S3). Among the 27 included studies, 15 evaluated the efficacy of SGLT2i, RASi, or ARNI in patients with HFpEF. Specifically, SGLT2i studies included empagliflozin (4 studies) (Abraham et al., 2021; Anker et al., 2021; Voors et al., 2022; Ovchinnikov et al., 2025), dapagliflozin (3 studies) (Singh et al., 2020; Nassif et al., 2021; McMurray et al., 2024), and sotagliflozin (1 study) (Szarek et al., 2021); 4 studies involved RASi (Yusuf et al., 2003; Cleland et al., 2006; Massie et al., 2008; Parthasarathy et al., 2009) and 3 involved ARNI (Solomon et al., 2019; Ledwidge et al., 2023; Mentz et al., 2023). In addition, 2 studies focused on patients with HFmrEF (Solomon et al., 2012; Bhatt et al., 2021), evaluating sotagliflozin and ARNI, respectively. The remaining 10 studies included populations with HFmrEF or HFpEF, involving SGLT2i (dapagliflozin, 2 studies (Solomon et al., 2022; Yang et al., 2022); canagliflozin, 1 study (Spertus et al., 2022); empagliflozin, 1 study (Tromp et al., 2024)), finerenone (5 studies) (Solomon et al., 2024; Chimura et al., 2025; Matsumoto et al., 2025; Vaduganathan et al., 2025; Yang et al., 2025), and RASi (1 study) (Zi et al., 2003).

**TABLE 1:** Baseline characteristics of the included studies.

Study	Study design	Patients	Sample size (E.G.,/CG)	Age (year)	Male (%)	Intervention of, E.G.,	Intervention of CG	Outcomes
Abraham et al. (2021)	RCT	HFpEF	157/158	74.52	56.83	Empagliflozin 10 mg	Placebo	a,c,f
McMurray et al. (2024)	RCT	HFpEF	253/251	73.05	63.50	Dapagliflozin 10 mg	Placebo	a,f
Nassif et al. (2021)	RCT	HFpEF	162/162	70.01	43.21	Dapagliflozin 10 mg	Placebo	c,d,e,f
Ovchinnikov et al. (2025)	RCT	HFpEF	35/35	67.05	37.02	Empagliflozin 10 mg	Placebo	f
Solomon et al. (2022)	RCT	HFmrEF/HFpEF	3,131/3,132	71.65	56.14	Dapagliflozin 10 mg once daily	Placebo	a,b,c,d,e,f
Solomon et al. (2024)	RCT	HFmrEF/HFpEF	3,003/2,998	71.95	54.47	Finerenone 20/40 mg once daily	Placebo	a,b,c,d,f
Spertus et al. (2022)	RCT	HFmrEF/HFpEF	222/226	63.42	55.01	Canagliflozin 100 mg	Placebo	a,b,d,f
Tromp et al. (2024)	RCT	HFmrEF/HFpEF	76/93	74.09	68.35	Empagliflozin 10 mg once daily	Placebo	a,b,c,d,f
Vaduganathan et al. (2025)	RCT	HFmrEF/HFpEF	2,610/2,574	72.00	54.54	Finerenone, eGFR ≤60 mL/min/1.73 m^2^ 20 mg; eGFR >60 mL/min/1.73 m^2^ 40 mg	Placebo	a,b,c,d,e,f
Chimura et al. (2025)	RCT	HFmrEF/HFpEF	2,836/2,829	73.11	54.70	Finerenone 20–40 mg	Placebo	a,b,d
Matsumoto et al. (2025)	RCT	HFmrEF/HFpEF	2,992/2,992	72.00	54.48	Finerenone, eGFR ≤60 mL/min/1.73 m^2^ 10 mg–20 mg; eGFR >60 mL/min/1.73 m^2^ 20–40 mg	Placebo	a,b,d
Yang et al. (2025)	RCT	HFmrEF/HFpEF	2,995/2,991	72.01	54.49	finerenone, eGFR ≤60 mL/min/1.73 m^2^ 10 mg-20 mg once per day; eGFR >60 mL/min/1.73 m^2^ 20 mg-40 mg once per day	Placebo	a,b,d
Anker et al. (2021)	RCT	HFpEF	2,997/2,991	71.85	55.30	Empagliflozin 10 mg once daily	Placebo	a,c,d,e
Voors et al. (2022)	RCT	HFpEF	265/265	71.12	66.02	Empagliflozin 10 mg once daily	Placebo	a,b,c,f
Szarek et al. (2021)	RCT	HFpEF	608/614	69.50	66.28	Sotagliflozin 200–400 mg once daily	Placebo	a,e
Bhatt et al. (2021)	RCT	HFmrEF	608/614	69.54	62.84	Sotagliflozin	Placebo	a,b,c,d,e,f
Yang et al. (2022)	RCT	HFmrEF/HFpEF	2,996/2,966	71.84	55.50	Dapagliflozin 10 mg daily	Placebo	a,b,d,e
Singh et al. (2020)	RCT	HFpEF	28/28	67.13	66.12	Dapagliflozin 10 mg daily	Placebo	a,d,e
Massie et al. (2008)	RCT	HFpEF	2067/2061	72.01	39.66	Irbesartan 300 mg	Placebo	a,b,c,d,e,f
Cleland et al. (2006)	RCT	HFpEF	424/426	75.03	44.53	Perindopril 4 mg	Placebo	a,b,e,f
Yusuf et al. (2003)	RCT	HFpEF	1,514/1,509	67.15	59.95	Candesartan 32 mg once daily	Placebo	a,e,f
Parthasarathy et al. (2009)	RCT	HFpEF	68/82	62.15	50.02	Valsartan 320 mg	Placebo	f
Zi et al. (2003)	RCT	HFmrEF/HFpEF	36/38	77.51	64.87	Quinapril 40 mg	Placebo	a,b,e,f
Ledwidge et al. (2023)	RCT	HFpEF	122/128	72.03	61.62	Sacubitril/valsartan 200 mg twice daily	Valsartan200 mg twice daily	a,c,f
Mentz et al. (2023)	RCT	HFpEF	233/233	71.50	48.07	Sacubitril/valsartan 97/103 mg twice daily	Valsartan 160 mg twice daily	a,b,c,e,f
Solomon et al. (2019)	RCT	HFpEF	2,407/2,389	72.75	51.69	Sacubitril/valsartan 97/103 mg twice daily	Valsartan 160 mg twice daily	a,b,c,d,e,f
Solomon et al. (2012)	RCT	HFmrEF	149/152	71.05	43.52	Sacubitril/valsartan 200 mg twice daily	Valsartan 160 mg twice daily	a,b,c,f

Baseline characteristics of the included studies. a: Cardiovascular death; b: Worsening heart failure events; c: Composite renal outcome; d: All-cause mortality; e: Total heart failure hospitalizations; f: Adverse events; EG: experimental group; CG: control group; eGFR: estimated glomerular filtration rate; RCT: randomized controlled trial.

### 3.2 Risk of bias assessment

**FIGURE 2:**
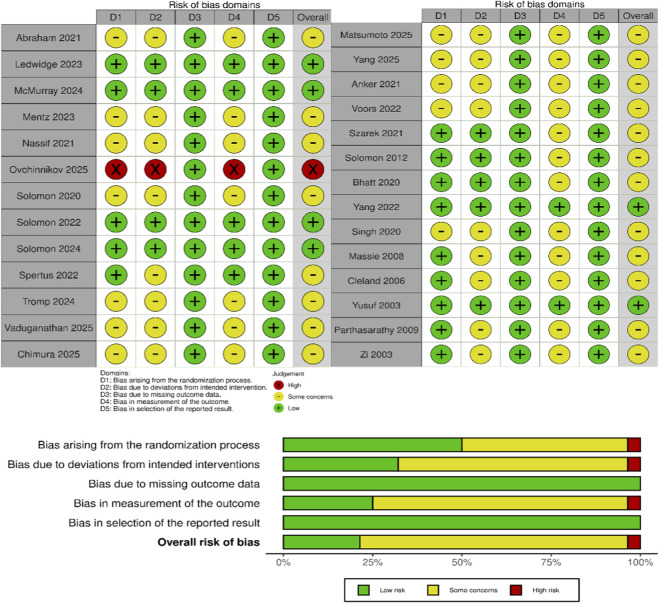
Risk of bias.

The risk of bias of the 27 included RCTs was assessed using RoB 2 (Sterne et al., 2019), with the results summarized in Figure 2. With respect to allocation concealment, eight studies were judged to be at low risk of bias, as they employed methods such as computer-generated randomization or centralized randomization systems; 18 studies were rated as having some concerns owing to insufficient information regarding the concealment process; and one study was classified as high risk because allocation concealment was not implemented.

### 3.3 Clinical outcomes analysis

#### 3.3.1 Network evidence structure

**FIGURE 3:**
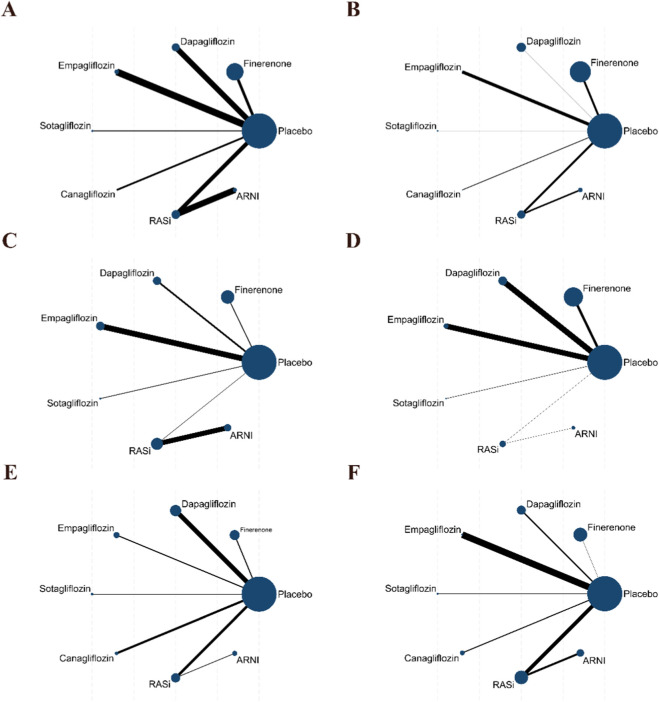
Network plot. **(A)** Cardiovascular death; **(B)** Worsening heart failure events; **(C)** Composite renal outcome; **(D)** All-cause mortality; **(E)** Total heart failure hospitalizations; **(F)** Adverse events.

A total of 107 outcome data points were extracted. The network evidence structure was primarily based on placebo-controlled comparisons, while also including a limited number of direct comparisons between drugs. The network plot was constructed to illustrate the comparisons involved in the network meta-analysis. As no closed loops were present in the network, comparisons among the eight interventions were performed using a consistency model. In the network plot, blue circles represent the different interventions, with the size of each circle corresponding to the sample size of the respective intervention. Lines connecting these circles indicate studies directly comparing the corresponding interventions, and the thickness of these lines reflects the number of studies contributing to each comparison (Figure 3).

#### 3.3.2 Cardiovascular death

**FIGURE 4:**
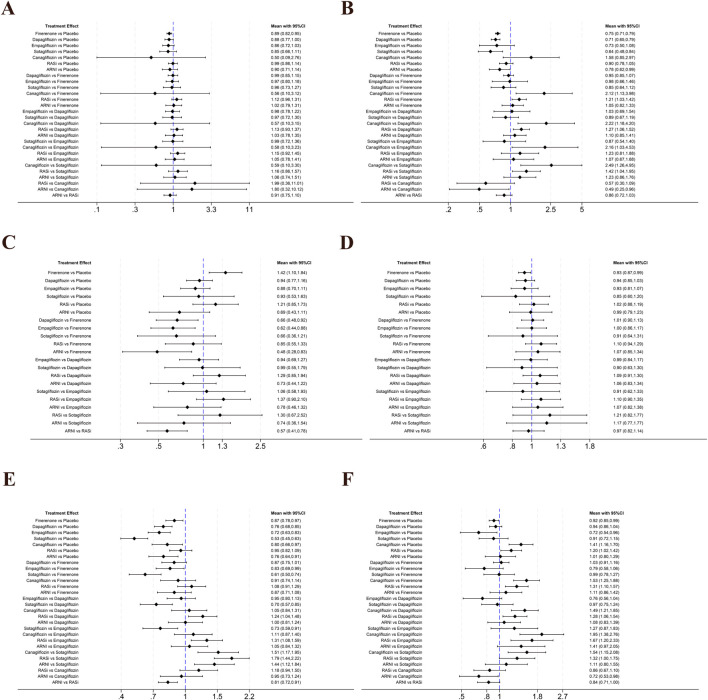
Pairwise comparison forest plot. **(A)** Cardiovascular death; **(B)** Worsening heart failure events; **(C)** Composite renal outcome; **(D)** All-cause mortality; **(E)** Total heart failure hospitalizations; **(F)** Adverse events.

**FIGURE 5:**
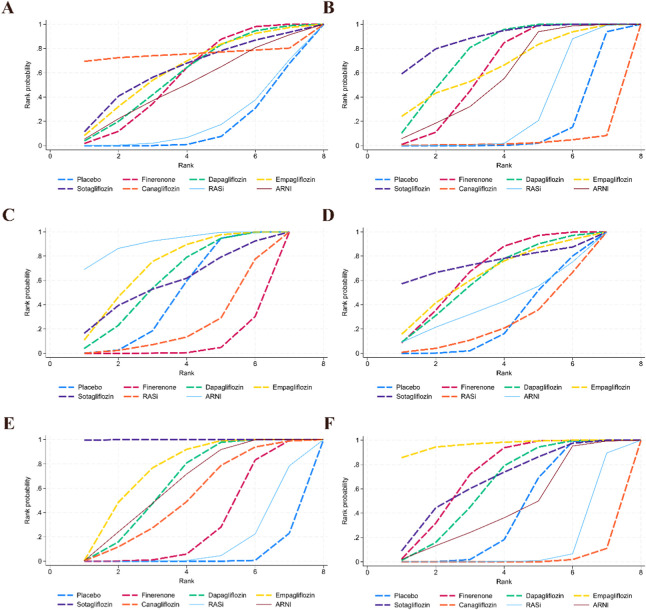
Cumulative ranking curves. **(A)** Cardiovascular death; **(B)** Worsening heart failure events; **(C)** Composite renal outcome; **(D)** All-cause mortality; **(E)** Total heart failure hospitalizations; **(F)** Adverse events.

A total of 24 studies contributed data on CV death, involving finerenone, empagliflozin, dapagliflozin, canagliflozin, sotagliflozin, RASi, and ARNI; the network structure of the interventions is shown in Figure 3. Compared with placebo, only finerenone was associated with a lower risk of CV death (OR = 0.89, 95% CI 0.82–0.95), whereas no statistically significant differences were observed for the other interventions versus placebo. No statistically significant differences were observed in indirect comparisons between interventions (Figure 4 and Supplementary Table S4). The SUCRA curves for each outcome are shown in Figure 5, and the forest plots and SUCRA ranking results are presented in Supplementary Figures S1, S2.

#### 3.3.3 Worsening HF events

A total of 17 studies contributed data on worsening HF events, including finerenone, empagliflozin, dapagliflozin, canagliflozin, sotagliflozin, RASi, and ARNI; the network structure is shown in Figure 3. Compared with placebo, sotagliflozin (OR = 0.64, 95% CI 0.48–0.84), dapagliflozin (OR = 0.71, 95% CI 0.65–0.79), finerenone (OR = 0.75, 95% CI 0.71–0.79), and ARNI (OR = 0.78, 95% CI 0.62–0.99) were associated with a lower risk, whereas no statistically significant differences were observed for empagliflozin, canagliflozin, and RASi versus placebo. Comparisons between interventions were primarily based on indirect evidence. Compared with finerenone, canagliflozin (OR = 2.12, 95% CI 1.13–3.98) and RASi (OR = 1.21, 95% CI 1.03–1.42) were associated with a higher risk. Within SGLT2i, canagliflozin was associated with a higher risk than dapagliflozin (OR = 2.22, 95% CI 1.18–4.20), empagliflozin (OR = 2.16, 95% CI 1.03–4.53), and sotagliflozin (OR = 2.49, 95% CI 1.26–4.95). In addition, RASi was associated with a higher risk than sotagliflozin (OR = 1.42, 95% CI 1.04–1.95), whereas ARNI was associated with a lower risk than canagliflozin (OR = 0.49, 95% CI 0.25–0.96). No statistically significant differences were observed for the remaining comparisons (Figure 4 and Supplementary Table S5). The SUCRA curves for each outcome are shown in Figure 5, and the forest plots and SUCRA ranking results are presented in Supplementary Figures S1, S2.

#### 3.3.4 Composite renal outcome

A total of 14 studies contributed data on composite renal outcomes, including finerenone, empagliflozin, dapagliflozin, sotagliflozin, RASi, and ARNI; the network structure is shown in Figure 3. Compared with placebo, finerenone was associated with a higher risk of composite renal outcomes (OR = 1.42, 95% CI 1.10–1.84), whereas no statistically significant differences were observed for the other interventions versus placebo. Comparisons between interventions were primarily based on indirect evidence. Compared with finerenone, dapagliflozin (OR = 0.66, 95% CI 0.48–0.92), empagliflozin (OR = 0.62, 95% CI 0.44–0.88), and ARNI (OR = 0.48, 95% CI 0.28–0.83) were associated with a lower risk. In addition, ARNI was associated with a lower risk than RASi (OR = 0.57, 95% CI 0.41–0.78). No statistically significant differences were observed for sotagliflozin or RASi compared with finerenone (Figure 4 and Supplementary Table S6). The SUCRA curves for each outcome are shown in Figure 5, and the forest plots and SUCRA ranking results are presented in Supplementary Figures S1, S2.

#### 3.3.5 All-cause mortality

A total of 15 studies contributed data on all-cause mortality, involving finerenone, empagliflozin, dapagliflozin, sotagliflozin, RASi, and ARNI; the network structure of these interventions is shown in Figure 3. Compared with placebo, only finerenone was associated with a lower risk of all-cause mortality (OR = 0.93, 95% CI 0.87–0.99), whereas no statistically significant differences were observed for the other interventions versus placebo. No statistically significant differences were observed in indirect comparisons between interventions (Figure 4 and Supplementary Table S7). The SUCRA curves for each outcome are shown in Figure 5, and the forest plots and SUCRA ranking results are presented in Supplementary Figures S1, S2.

#### 3.3.6 Total heart failure hospitalizations

A total of 17 studies contributed data on total HF hospitalizations, including finerenone, empagliflozin, dapagliflozin, canagliflozin, sotagliflozin, RASi, and ARNI; the network structure is shown in Figure 3. Compared with placebo, sotagliflozin (OR = 0.53, 95% CI 0.45–0.63), empagliflozin (OR = 0.72, 95% CI 0.63–0.83), dapagliflozin (OR = 0.76, 95% CI 0.68–0.85), ARNI (OR = 0.76, 95% CI 0.64–0.91), canagliflozin (OR = 0.80, 95% CI 0.66–0.97), and finerenone (OR = 0.87, 95% CI 0.78–0.97) were associated with a lower risk of total HF hospitalizations, whereas no statistically significant differences were observed for RASi versus placebo. Comparisons between interventions were primarily based on indirect evidence. Compared with finerenone, sotagliflozin (OR = 0.61, 95% CI 0.50–0.74) and empagliflozin (OR = 0.83, 95% CI 0.69–0.99) were associated with a lower risk. Within SGLT2i, sotagliflozin was associated with a lower risk compared with dapagliflozin (OR = 0.70, 95% CI 0.57–0.85) and empagliflozin (OR = 0.73, 95% CI 0.59–0.91), whereas no statistically significant differences were observed for the remaining comparisons (Figure 4 and Supplementary Table S8). The SUCRA curves for each outcome are shown in Figure 5, and the forest plots and SUCRA ranking results are presented in Supplementary Figures S1, S2.

#### 3.3.7 Adverse events

A total of 20 studies contributed data on adverse events, including finerenone, empagliflozin, dapagliflozin, canagliflozin, sotagliflozin, RASi, and ARNI; the network structure is shown in Figure 3. Compared with placebo, finerenone (OR = 0.92, 95% CI 0.85–0.99) and empagliflozin (OR = 0.72, 95% CI 0.54–0.96) were associated with a lower risk of adverse events, whereas canagliflozin (OR = 1.41, 95% CI 1.16–1.70) and RASi (OR = 1.20, 95% CI 1.02–1.42) were associated with a higher risk, and no statistically significant differences were observed for the remaining interventions versus placebo. Comparisons between interventions were primarily based on indirect evidence. Compared with finerenone, canagliflozin (OR = 1.53, 95% CI 1.25–1.88) and RASi (OR = 1.31, 95% CI 1.10–1.57) were associated with a higher risk, whereas no statistically significant differences were observed for dapagliflozin, empagliflozin, sotagliflozin, or ARNI versus finerenone. Within SGLT2i, canagliflozin was associated with a higher risk than dapagliflozin (OR = 1.49, 95% CI 1.21–1.85), empagliflozin (OR = 1.95, 95% CI 1.38–2.76), and sotagliflozin (OR = 1.54, 95% CI 1.15–2.08), whereas no statistically significant differences were observed for the remaining comparisons. In addition, ARNI was associated with a lower risk than canagliflozin (OR = 0.72, 95% CI 0.53–0.98), whereas no statistically significant differences were observed compared with RASi (OR = 0.84, 95% CI 0.71–1.00) (Figure 4 and Supplementary Table S9). The SUCRA curves for each outcome are shown in Figure 5, and the forest plots and SUCRA ranking results are presented in Supplementary Figures S1, S2.

## 4 Sensitivity analyses and bias assessment

**FIGURE 6:**
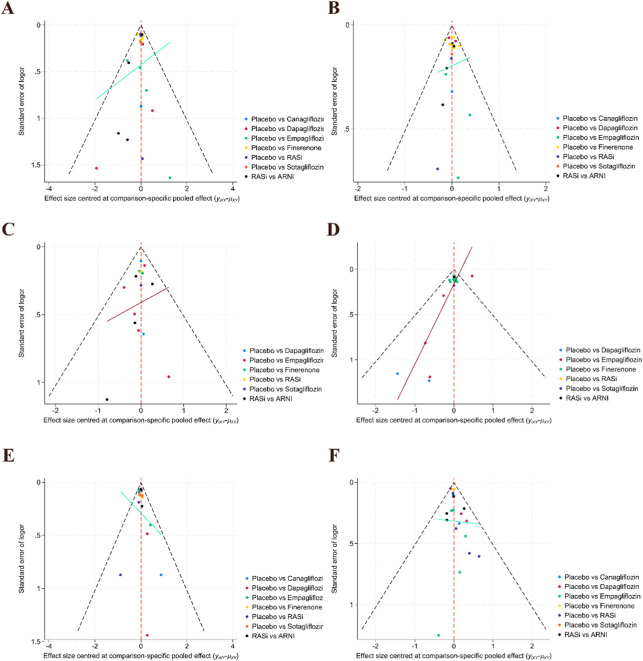
Funnel plot. **(A)** Cardiovascular death; **(B)** Worsening heart failure events; **(C)** Composite renal outcome; **(D)** All-cause mortality; **(E)** Total heart failure hospitalizations; **(F)** Adverse events.

To assess the robustness of the findings, sensitivity analyses were performed. After excluding studies at high risk of bias and those with small sample sizes, the direction of effects and overall results across outcomes remained materially unchanged (Supplementary Figures S3–S5), indicating a certain degree of robustness. Funnel plots were generated using Stata version 18.0 MP to explore potential publication bias (Figure 6). Overall, most studies were distributed around the line of no effect, and no clear evidence of systematic asymmetry was observed; however, for some outcomes with a limited number of studies, several points were located at the margins or lower part of the funnel plots, suggesting the possible presence of small-study effects in certain cases. Given the limited number of studies and the distribution of sample sizes, the assessment of publication bias remains subject to inherent limitations.

## 5 Discussion

### 5.1 Overall findings

This analysis included 27 RCTs, encompassing 65,929 patients. The findings suggest that, compared with placebo, several interventions were associated with reduced risks of CV death, worsening HF events, all-cause mortality, total HF hospitalizations, and adverse events, whereas finerenone may be associated with an increased risk of composite renal outcomes. While prior studies have largely focused on evaluating outcomes of SGLT2i, ARNI, and RASi in patients with HFpEF (Ji et al., 2023), evidence regarding renal outcomes associated with finerenone in individuals with HFpEF or HFmrEF remains comparatively limited. Overall, in the context of limited direct comparisons among finerenone, SGLT2i, RASi, and ARNI, the present study, by incorporating evidence from the recently published FINEARTS-HF trial, provides a systematic comparative assessment of clinical outcomes and safety across these therapies in patients with HFpEF and HFmrEF.

### 5.2 Finerenone and HF

Previous studies indicate that efficacy and safety outcomes in patients with HF are commonly used to reflect disease progression (Lin et al., 2022). In the present study, compared with placebo, finerenone treatment in patients with HFpEF or HFmrEF was associated with an overall reduction in the risk of relevant outcomes, with more pronounced effects observed for worsening HF events (OR = 0.75, 95% CI 0.71–0.79) and total HF hospitalizations (OR = 0.87, 95% CI 0.78–0.97). Li et al. reported that HFpEF and HFmrEF share similar pathophysiological features, with potential common mechanisms involving inflammatory responses, cardiomyocyte injury, endothelial dysfunction, myocardial fibrosis, and mitochondrial dysfunction (Li et al., 2021). HFpEF is primarily associated with left ventricular diastolic dysfunction (Budde et al., 2022), chronic inflammation, and microvascular dysfunction, whereas HFmrEF is more commonly linked to ischaemic heart disease and mild impairment of left ventricular systolic function (Hsu et al., 2017). Tesch suggested that mineralocorticoid receptor (MR) signalling may contribute to myocardial remodelling and to the development and progression of HF by promoting inflammation, oxidative stress, and myocardial fibrosis (Tesch and Young, 2017). Finerenone, a highly selective non-steroidal MRA, inhibits MR-mediated pathological processes related to inflammation, oxidative stress, and fibrosis (Ramadan et al., 2025). Previous meta-analyses have indicated that finerenone treatment is associated with reduced risks of HF hospitalization and all-cause mortality (Rivera-Martinez et al., 2026). Furthermore, a secondary analysis of the FINEARTS-HF trial demonstrated that finerenone reduced HF-related events and was associated with a trend towards lower CV death (Docherty et al., 2025). In addition, a retrospective study reported a reduced risk of all-cause mortality among patients with HFpEF or HFmrEF receiving finerenone (Calcagno et al., 2025). Taken together, whereas prior evidence has largely been derived from populations with type 2 diabetes and chronic kidney disease, the present study focuses on patients with HFpEF or HFmrEF, thereby extending the evidence base for finerenone in this population.

### 5.3 Composite renal outcomes of finerenone

In the present study, compared with placebo, finerenone may be associated with an increased risk of composite renal outcomes. However, the FIDELIO-DKD and FIGARO-DKD trials showed that, in patients with type 2 diabetes and chronic kidney disease, finerenone was associated with a reduced risk of composite renal outcomes (Bakris et al., 2020; Agarwal et al., 2021). This apparent inconsistency may reflect differences in study populations, as the present analysis included patients with HFpEF or HFmrEF, whereas the aforementioned trials primarily enrolled individuals with type 2 diabetes and chronic kidney disease, whose baseline renal risk profiles differ. In addition, differences in baseline renal function, the degree of proteinuria, and the underlying risk of renal events across studies may further contribute to this discrepancy. Variations in follow-up duration may also influence the cumulative incidence of renal events, thereby affecting comparisons of composite renal outcomes. In the FINEARTS-HF trial, 75 composite renal events occurred in the finerenone group compared with 55 in the placebo group (HR = 1.33, 95% CI 0.94–1.89), with no statistically significant difference observed between groups, suggesting that no clear reduction in the risk of composite renal outcomes was evident in this population (Solomon et al., 2024). Previous studies have employed varying thresholds for declines in estimated glomerular filtration rate (eGFR) (≥40%, ≥50%, or ≥57%), combined with renal replacement therapy or renal death to define composite endpoints, indicating heterogeneity in the definition of composite renal outcomes across studies (Perkovic et al., 2020; Heerspink et al., 2023). Furthermore, patients with HFpEF or HFmrEF commonly receive background therapies, including SGLT2i, RASi, and ARNI, which may confer renal protective effects to varying degrees and thereby influence the observed effects of finerenone. Given that the number of large randomized controlled trials of finerenone remains limited and that evidence regarding composite renal outcomes across different populations is relatively sparse, and considering that the present findings are primarily derived from indirect comparisons, the effect of finerenone on composite renal outcomes in patients with HFpEF or HFmrEF should be interpreted with caution.

### 5.4 Safety of SGLT2i, ARNI, and RASi

This analysis suggests that differences may exist in outcomes associated with SGLT2i, ARNI, and RASi in patients with HFpEF or HFmrEF. Compared with placebo, SGLT2i were associated with lower risks of worsening HF events and total HF hospitalizations, with both sotagliflozin and dapagliflozin demonstrating statistically significant associations, whereas ARNI were also associated with lower risks of worsening HF events and composite renal outcomes. In contrast, RASi did not show statistically significant differences in worsening HF events or total HF hospitalizations compared with placebo, but were associated with a higher risk of adverse events. Previous studies likewise indicate that SGLT2i confer benefits in reducing the risk of HF hospitalization in patients with HFpEF or HFmrEF, with ARNI also demonstrating clinical benefit (Xiang et al., 2022). Although the present study compared different therapeutic strategies, these findings are primarily derived from indirect comparisons and therefore require further validation. In addition, although evidence from RCTs for ertugliflozin in patients with HFpEF or HFmrEF remains limited, existing studies suggest potential benefits in outcomes such as HF hospitalization (Pescariu et al., 2024).

### 5.5 Implications

The present study, conducted in a population with HFpEF or HFmrEF, provides a systematic comparison of finerenone, SGLT2i, ARNI, and RASi with respect to clinical outcomes and safety. Furthermore, building on evidence derived from previous RCTs, this analysis incorporates data from the FINEARTS-HF trial to evaluate the therapeutic effects of finerenone in patients with HFpEF or HFmrEF. The 2025 JCS/JHFS heart failure guidelines list finerenone as a treatment that may be considered in patients with HFpEF or HFmrEF (Kitai et al., 2025), with the aim of reducing CV death and worsening HF events. These guidelines recommend that, in patients with symptomatic chronic HF and a left ventricular ejection fraction of ≥40%, finerenone may be considered on top of standard therapy. The ESC heart failure guidelines indicate that SGLT2i are recommended in patients with HFmrEF or HFpEF to reduce the risks of HF hospitalization and CV death, whereas evidence for ARNI and RASi in this population remains limited and their use is primarily established in patients with HFrEF (McDonagh et al., 2024). The present findings are consistent with current guideline recommendations and provide exploratory supplementary evidence from a network meta-analysis.

### 5.6 Limitations

Several limitations of this study should be acknowledged. First, as most included studies were placebo-controlled, direct comparative evidence between active interventions remains limited, such that the majority of comparisons rely on indirect evidence, which may influence the results. In this context, differences across studies in patient characteristics and background therapies may affect comparability, and the inconsistent reporting of such information across studies may further introduce uncertainty into the indirect comparisons. Second, the overlap of confidence intervals in some comparisons suggests that differences between interventions remain uncertain. Third, evidence for composite renal outcomes is largely derived from the FINEARTS-HF trial, and variations in the definition of composite renal endpoints across studies may affect the robustness of these comparisons. Finally, heterogeneity in patient characteristics, follow-up duration, and outcome definitions across the included studies may introduce clinical heterogeneity. The findings of this study are therefore intended to provide a comparative reference for different therapies in patients with HFpEF or HFmrEF and should be regarded as exploratory, with clinical decision-making best informed by integrating direct evidence from comparisons with placebo.

## 6 Conclusion

In patients with HFpEF or HFmrEF, finerenone was associated with reduced risks of CV death and worsening HF events compared with placebo. Comparisons between interventions were primarily based on indirect evidence. With respect to total HF hospitalizations, sotagliflozin and empagliflozin were associated with lower risks than finerenone. No statistically significant differences were observed between interventions for CV death or all-cause mortality. For composite renal outcomes, ARNI, empagliflozin, and dapagliflozin were associated with lower risks compared with finerenone, whereas evidence for finerenone remains uncertain. Regarding adverse events, canagliflozin and RASi were associated with higher risks. Overall, although finerenone suggests potential clinical benefit in patients with HFpEF or HFmrEF, these findings, which are largely derived from indirect comparisons, require further validation through direct comparative studies.
