# Supplementary material for: Comparative clinical outcomes and safety of finerenone, SGLT2 inhibitors, RAS inhibitors and ARNI in heart failure with preserved or mildly reduced ejection fraction: a systematic review and network meta-analysis
Source: Front Pharmacol. 2026 May 15;17:1813205. doi: 10.3389/fphar.2026.1813205 (PMC13219348; doi:10.3389/fphar.2026.1813205)
Supplement: Supplementary file 1 [file DataSheet1.docx]

**Supplementary Information**

**Table S1. PRISMA 2020 checklist**

| **Section and Topic** | **Item #** | **Checklist item** | **Location where item is reported** |
| --- | --- | --- | --- |
| **TITLE** | | |  |
| Title | 1 | Identify the report as a systematic review. | Title page |
| **ABSTRACT** | | |  |
| Abstract | 2 | See the PRISMA 2020 for Abstracts checklist. | Abstract |
| **INTRODUCTION** | | |  |
| Rationale | 3 | Describe the rationale for the review in the context of existing knowledge. | Introduction |
| Objectives | 4 | Provide an explicit statement of the objective(s) or question(s) the review addresses. | Introduction |
| **METHODS** | | |  |
| Eligibility criteria | 5 | Specify the inclusion and exclusion criteria for the review and how studies were grouped for the syntheses. | Methods 2.3 |
| Information sources | 6 | Specify all databases, registers, websites, organisations, reference lists and other sources searched or consulted to identify studies. Specify the date when each source was last searched or consulted. | Methods 2.2 |
| Search strategy | 7 | Present the full search strategies for all databases, registers and websites, including any filters and limits used. | Methods 2.2 |
| Selection process | 8 | Specify the methods used to decide whether a study met the inclusion criteria of the review, including how many reviewers screened each record and each report retrieved, whether they worked independently, and if applicable, details of automation tools used in the process. | Methods 2.4 |
| Data collection process | 9 | Specify the methods used to collect data from reports, including how many reviewers collected data from each report, whether they worked independently, any processes for obtaining or confirming data from study investigators, and if applicable, details of automation tools used in the process. | Methods 2.4 |
| Data items | 10a | List and define all outcomes for which data were sought. Specify whether all results that were compatible with each outcome domain in each study were sought (e.g. for all measures, time points, analyses), and if not, the methods used to decide which results to collect. | Methods 2.5 |
|  | 10b | List and define all other variables for which data were sought (e.g. participant and intervention characteristics, funding sources). Describe any assumptions made about any missing or unclear information. | Methods 2.4 |
| Study risk of bias assessment | 11 | Specify the methods used to assess risk of bias in the included studies, including details of the tool(s) used, how many reviewers assessed each study and whether they worked independently, and if applicable, details of automation tools used in the process. | Methods 2.6 |
| Effect measures | 12 | Specify for each outcome the effect measure(s) (e.g. risk ratio, mean difference) used in the synthesis or presentation of results. | Methods 2.7 |
| Synthesis methods | 13a | Describe the processes used to decide which studies were eligible for each synthesis (e.g. tabulating the study intervention characteristics and comparing against the planned groups for each synthesis (item #5)). | Methods 2.3 |
|  | 13b | Describe any methods required to prepare the data for presentation or synthesis, such as handling of missing summary statistics, or data conversions. | Methods 2.7 |
|  | 13c | Describe any methods used to tabulate or visually display results of individual studies and syntheses. | Methods 2.7 |
|  | 13d | Describe any methods used to synthesize results and provide a rationale for the choice(s). If meta-analysis was performed, describe the model(s), method(s) to identify the presence and extent of statistical heterogeneity, and software package(s) used. | Methods 2.7 |
|  | 13e | Describe any methods used to explore possible causes of heterogeneity among study results (e.g. subgroup analysis, meta-regression). | Methods 2.7 |
|  | 13f | Describe any sensitivity analyses conducted to assess robustness of the synthesized results. | Methods 2.7 |
| Reporting bias assessment | 14 | Describe any methods used to assess risk of bias due to missing results in a synthesis (arising from reporting biases). | Methods 2.7 |
| Certainty assessment | 15 | Describe any methods used to assess certainty (or confidence) in the body of evidence for an outcome. | Methods 2.7 |
| **RESULTS** | | |  |
| Study selection | 16a | Describe the results of the search and selection process, from the number of records identified in the search to the number of studies included in the review, ideally using a flow diagram. | Results 3.1 |
|  | 16b | Cite studies that might appear to meet the inclusion criteria, but which were excluded, and explain why they were excluded. | Results 3.1 |
| Study characteristics | 17 | Cite each included study and present its characteristics. | Results 3.1 |
| Risk of bias in studies | 18 | Present assessments of risk of bias for each included study. | Results 3.2 |
| Results of individual studies | 19 | For all outcomes, present, for each study: (a) summary statistics for each group (where appropriate) and (b) an effect estimate and its precision (e.g. confidence/credible interval), ideally using structured tables or plots. | Results 3.3 |
| Results of syntheses | 20a | For each synthesis, briefly summarise the characteristics and risk of bias among contributing studies. | Results 3.3 |
|  | 20b | Present results of all statistical syntheses conducted. If meta-analysis was done, present for each the summary estimate and its precision (e.g. confidence/credible interval) and measures of statistical heterogeneity. If comparing groups, describe the direction of the effect. | Results 3.3 |
|  | 20c | Present results of all investigations of possible causes of heterogeneity among study results. | Results 4 |
|  | 20d | Present results of all sensitivity analyses conducted to assess the robustness of the synthesized results. | Results 4 |
| Reporting biases | 21 | Present assessments of risk of bias due to missing results (arising from reporting biases) for each synthesis assessed. | Results 4 |
| Certainty of evidence | 22 | Present assessments of certainty (or confidence) in the body of evidence for each outcome assessed. | Discussion 4.5 |
| **DISCUSSION** | | |  |
| Discussion | 23a | Provide a general interpretation of the results in the context of other evidence. | Discussion 4.1 |
|  | 23b | Discuss any limitations of the evidence included in the review. | Discussion 4.5 |
|  | 23c | Discuss any limitations of the review processes used. | Discussion 4.5 |
|  | 23d | Discuss implications of the results for practice, policy, and future research. | Discussion 4.5 |
| **OTHER INFORMATION** | | |  |
| Registration and protocol | 24a | Provide registration information for the review, including register name and registration number, or state that the review was not registered. | Methods 2.1 |
|  | 24b | Indicate where the review protocol can be accessed, or state that a protocol was not prepared. | Methods 2.1 |
|  | 24c | Describe and explain any amendments to information provided at registration or in the protocol. | Methods 2.1 |
| Support | 25 | Describe sources of financial or non-financial support for the review, and the role of the funders or sponsors in the review. | Funding |
| Competing interests | 26 | Declare any competing interests of review authors. | Conflict of Interest |
| Availability of data, code and other materials | 27 | Report which of the following are publicly available and where they can be found: template data collection forms; data extracted from included studies; data used for all analyses; analytic code; any other materials used in the review. | Data Availability Statement |

**Table S2. search strategy**

**Search strategy of PubMed**

| NO. | Search Details | Results |
| --- | --- | --- |
| #9 | (#1 OR #3) AND (#2 OR #4 OR #5 OR #6 OR #7) AND #8 | 1693 |
| #8 | (clinical drug trial) OR (major clinical trial) OR (trial, clinical) OR (clinical trial) | 2,430913 |
| #7 | (((((((((((((((((((((((((angiotensin‐converting enzyme inhibitor) OR (angiotensin receptor antagonist)) OR (RAS inhibitor)) OR (renin‐angiotensin system inhibitor )) OR (angiotensin receptor blockers)) OR (angiotensin receptor‐neprilysin inhibitor)) OR (ACE inhibitor)) OR （ARN inhibitor)) OR (ACEI)) OR (ARNI)) OR (ARB)) OR (Captopril)) OR (Enalapril)) OR (Cilazapril)) OR (fosinopril )) OR (lisinopril)) OR (perindopril)) OR (ramipril)) OR (candesartan)) OR (irbesartan)) OR (losartan)) OR (telmisartan)) OR (valsartan)) OR (Olmesartan)) OR (Eprosartan)) OR (sacubitril‐valsartan) | 130595 |
| #6 | ((Angiotensin receptor enkephalinase inhibitors) OR (Angiotensin receptor enkephalinase inhibitor)) OR (ARNI) | 2,695 |
| #5 | (((((((((((((((((((Sodium Glucose Transporter 2 Inhibitors) OR (SGLT-2 Inhibitors)) OR (SGLT 2 Inhibitors)) OR (SGLT-2 Inhibitor)) OR (SGLT 2 Inhibitor)) OR (Sodium-Glucose Transporter 2 Inhibitor)) OR (Sodium Glucose Transporter 2 Inhibitor)) OR (SGLT2 Inhibitor)) OR (Gliflozins)) OR (Gliflozin)) OR (SGLT2 Inhibitors)) OR (gliflozin)) OR (gliflozin derivative)) OR (gliflozins)) OR (SGLT2 inhibitor)) OR (SGLT2 inhibitors)) OR (sodium dependent glucose cotransporter 2 inhibitor)) OR (sodium glucose co-transporter 2 inhibitor)) OR (sodium-glucose transporter 2 inhibitors)) OR (sodium glucose cotransporter 2 inhibitor) | 18775 |
| #4 | (Finerenone) or (kerendia) | 906 |
| #3 | (((((((Diastolic Heart Failures) OR (Diastolic Heart Failure)) OR (Heart Failure, Preserved Ejection Fraction)) OR (Heart Failure, Normal Ejection Fraction)) OR (HF with preserved ejection fraction)) OR (HF-pEF)) OR (HFpEF )) OR (heart failure with preserved ejection fraction) | 38821 |
| #2 | "Sodium-Glucose Transporter 2 Inhibitors"[Mesh] | 9028 |
| #1 | "Heart Failure, Diastolic"[Mesh] | 894 |

**Search strategy of EMBASE**

| No. | Query | Results |
| --- | --- | --- |
| #11 | (#1 OR #2) AND (#3 OR #4 OR #5 OR #6 OR #7 OR #8) AND (#9 OR #10) | 954 |
| #10 | 'clinical drug trial':ti,ab,kw OR 'major clinical trial':ti,ab,kw OR 'trial, clinical':ti,ab,kw OR 'clinical trial':ti,ab,kw | 448167 |
| #9 | 'clinical trial'/exp | 2677990 |
| #8 | 'angiotensin‐converting enzyme inhibitor':ti,ab,kw OR 'angiotensin receptor antagonist':ti,ab,kw OR 'RAS inhibitor':ti,ab,kw OR 'renin‐angiotensin system inhibitor ':ti,ab,kw OR 'angiotensin receptor blockers':ti,ab,kw OR 'angiotensin receptor‐neprilysin inhibitor':ti,ab,kw OR 'ACE inhibitor':ti,ab,kw OR 'ARN inhibitor':ti,ab,kw OR 'ACEI':ti,ab,kw OR 'ARNI':ti,ab,kw OR 'ARB':ti,ab,kw OR 'Captopril':ti,ab,kw OR 'Enalapril':ti,ab,kw OR 'Cilazapril':ti,ab,kw OR 'fosinopril ':ti,ab,kw OR 'lisinopril':ti,ab,kw OR 'perindopril':ti,ab,kw OR 'ramipril':ti,ab,kw OR 'candesartan':ti,ab,kw OR 'irbesartan':ti,ab,kw OR 'losartan':ti,ab,kw OR 'telmisartan':ti,ab,kw OR 'valsartan':ti,ab,kw OR 'Olmesartan':ti,ab,kw OR 'Eprosartan':ti,ab,kw OR 'sacubitril‐valsartan':ti,ab,kw | 108996 |
| #7 | 'angiotensin receptor enkephalinase inhibitors':ti,ab,kw OR 'angiotensin receptor enkephalinase inhibitor':ti,ab,kw OR 'arni':ti,ab,kw | 2675 |
| #6 | 'sodium glucose transporter 2 inhibitors':ti,ab,kw OR 'sglt-2 inhibitors':ti,ab,kw OR 'sglt 2 inhibitors':ti,ab,kw OR 'sglt-2 inhibitor':ti,ab,kw OR 'sglt 2 inhibitor':ti,ab,kw OR 'sodium-glucose transporter 2 inhibitor':ti,ab,kw OR 'sodium glucose transporter 2 inhibitor':ti,ab,kw OR 'gliflozin':ti,ab,kw OR 'gliflozin derivative':ti,ab,kw OR 'gliflozins':ti,ab,kw OR 'sglt2 inhibitor':ti,ab,kw OR 'sglt2 inhibitors':ti,ab,kw OR 'sodium dependent glucose cotransporter 2 inhibitor':ti,ab,kw OR 'sodium glucose co-transporter 2 inhibitor':ti,ab,kw OR 'sodium-glucose transporter 2 inhibitors':ti,ab,kw OR 'sodium glucose cotransporter 2 inhibitor':ti,ab,kw | 20282 |
| #5 | 'sodium glucose cotransporter 2 inhibitor'/exp | 45006 |
| #4 | 'finerenone':ti,ab,kw OR 'kerendia':ti,ab,kw | 1340 |
| #3 | 'finerenone'/exp | 1826 |
| #2 | 'diastolic heart failures':ti,ab,kw OR 'diastolic heart failure':ti,ab,kw OR 'heart failure, preserved ejection fraction':ti,ab,kw OR 'heart failure, normal ejection fraction':ti,ab,kw OR 'hf with preserved ejection fraction':ti,ab,kw OR 'hf-pef':ti,ab,kw OR 'hfpef':ti,ab,kw OR 'heart failure with preserved ejection fraction':ti,ab,kw | 23992 |
| #1 | 'heart failure with preserved ejection fraction'/exp | 19915 |

**Search strategy of Cochrane Library**

| NO. | Search deatiles | Hits |
| --- | --- | --- |
| #1 | MeSH descriptor: [Heart Failure, Diastolic] explode all trees | 149 |
| #2 | MeSH descriptor: [Sodium-Glucose Transporter 2 Inhibitors] explode all trees | 1271 |
| #3 | (Diastolic Heart Failures):ti,ab,kw OR (Diastolic Heart Failure):ti,ab,kw OR (Heart Failure, Preserved Ejection Fraction):ti,ab,kw OR (Heart Failure, Normal Ejection Fraction):ti,ab,kw OR (HF with preserved ejection fraction):ti,ab,kw OR (HF-pEF):ti,ab,kw OR (HFpEF ):ti,ab,kw OR (heart failure with preserved ejection fraction):ti,ab,kw | 7686 |
| #4 | (Finerenone):ti,ab,kw or (kerendia):ti,ab,kw | 324 |
| #5 | (Sodium Glucose Transporter 2 Inhibitors):ti,ab,kw OR (SGLT-2 Inhibitors):ti,ab,kw OR (SGLT 2 Inhibitors):ti,ab,kw OR (SGLT-2 Inhibitor):ti,ab,kw OR (SGLT 2 Inhibitor):ti,ab,kw OR (Sodium-Glucose Transporter 2 Inhibitor):ti,ab,kw OR (Sodium Glucose Transporter 2 Inhibitor):ti,ab,kw OR (SGLT2 Inhibitor):ti,ab,kw OR (Gliflozins):ti,ab,kw OR (Gliflozin):ti,ab,kw OR (SGLT2 Inhibitors):ti,ab,kw OR (gliflozin):ti,ab,kw OR (gliflozin derivative):ti,ab,kw OR (gliflozins):ti,ab,kw OR (SGLT2 inhibitor):ti,ab,kw OR (SGLT2 inhibitors):ti,ab,kw OR (sodium dependent glucose cotransporter 2 inhibitor):ti,ab,kw OR (sodium glucose co-transporter 2 inhibitor):ti,ab,kw OR (sodium-glucose transporter 2 inhibitors):ti,ab,kw OR (sodium glucose cotransporter 2 inhibitor):ti,ab,kw | 4031 |
| #6 | (Angiotensin receptor enkephalinase inhibitors):ti,ab,kw OR (Angiotensin receptor enkephalinase inhibitor):ti,ab,kw OR (ARNI):ti,ab,kw | 384 |
| #7 | (clinical drug trial):ti,ab,kw OR (major clinical trial):ti,ab,kw OR (trial, clinical):ti,ab,kw OR (clinical trial):ti,ab,kw | 842232 |
| #8 | (angiotensin‐converting enzyme inhibitor):ti,ab,kw OR (angiotensin receptor antagonist):ti,ab,kw OR (RAS inhibitor):ti,ab,kw OR (renin‐angiotensin system inhibitor ):ti,ab,kw OR (angiotensin receptor blockers):ti,ab,kw OR (angiotensin receptor‐neprilysin inhibitor):ti,ab,kw OR (ACE inhibitor):ti,ab,kw OR (ARN inhibitor):ti,ab,kw OR (ACEI):ti,ab,kw OR (ARNI):ti,ab,kw OR (ARB):ti,ab,kw OR (Captopril):ti,ab,kw OR (Enalapril):ti,ab,kw OR (Cilazapril):ti,ab,kw OR (fosinopril ):ti,ab,kw OR (lisinopril):ti,ab,kw OR (perindopril):ti,ab,kw OR (ramipril):ti,ab,kw OR (candesartan):ti,ab,kw OR (irbesartan):ti,ab,kw OR (losartan):ti,ab,kw OR (telmisartan):ti,ab,kw OR (valsartan):ti,ab,kw OR (Olmesartan):ti,ab,kw OR (Eprosartan):ti,ab,kw OR (sacubitril‐valsartan):ti,ab,kw | 23489 |
| #9 | (#1 or #3) and (#2 or #4 or #5 or #6 or #7) and #8 | 975 |

**Search strategy of web of science**

| NO. | Search deatiles | Hits |
| --- | --- | --- |
| #1 | ((((((TS=(Diastolic Heart Failures) OR TS=(Diastolic Heart Failure)) OR TS=(Heart Failure, Preserved Ejection Fraction)) OR TS=(Heart Failure, Normal Ejection Fraction)) OR TS=(HF with preserved ejection fraction)) OR TS=(HF-pEF)) OR TS=(HFpEF )) OR TS=(heart failure with preserved ejection fraction) | 53280 |
| #2 | TS=(Finerenone) or TS=(kerendia) | 1289 |
| #3 | ((((((((((((((((((TS=(Sodium Glucose Transporter 2 Inhibitors) OR TS=(SGLT-2 Inhibitors)) OR TS=(SGLT 2 Inhibitors)) OR TS=(SGLT-2 Inhibitor)) OR TS=(SGLT 2 Inhibitor)) OR TS=(Sodium-Glucose Transporter 2 Inhibitor)) OR TS=(Sodium Glucose Transporter 2 Inhibitor)) OR TS=(SGLT2 Inhibitor)) OR TS=(Gliflozins)) OR TS=(Gliflozin)) OR TS=(SGLT2 Inhibitors)) OR TS=(gliflozin)) OR TS=(gliflozin derivative)) OR TS=(gliflozins)) OR TS=(SGLT2 inhibitor)) OR TS=(SGLT2 inhibitors)) OR TS=(sodium dependent glucose cotransporter 2 inhibitor)) OR TS=(sodium glucose co-transporter 2 inhibitor)) OR TS=(sodium-glucose transporter 2 inhibitors)) OR TS=(sodium glucose cotransporter 2 inhibitor) | 19300 |
| #4 | (TS=(Angiotensin receptor enkephalinase inhibitors) OR TS=(Angiotensin receptor enkephalinase inhibitor)) OR TS=(ARNI) | 1291 |
| #5 | ((((((((((((((((((((((((TS = (angiotensin‐converting enzyme inhibitor) OR TS = (angiotensin receptor antagonist)) OR TS = (RAS inhibitor)) OR TS = (renin‐angiotensin system inhibitor )) OR TS = (angiotensin receptor blockers)) OR TS = (angiotensin receptor‐neprilysin inhibitor)) OR TS = (ACE inhibitor)) OR TS = (ARN inhibitor)) OR TS = (ACEI)) OR TS = (ARNI)) OR TS = (ARB)) OR TS = (Captopril)) OR TS = (Enalapril)) OR TS = (Cilazapril)) OR TS = (fosinopril )) OR TS = (lisinopril)) OR TS = (perindopril)) OR TS = (ramipril)) OR TS = (candesartan)) OR TS = (irbesartan)) OR TS = (losartan)) OR TS = (telmisartan)) OR TS = (valsartan)) OR TS = (Olmesartan)) OR TS = (Eprosartan)) OR TS = (sacubitril‐valsartan) | 107434 |
| #6 | ((TS=(clinical drug trial) OR TS=(major clinical trial)) OR TS=(trial, clinical)) OR TS=(clinical trial) | 1006912 |
| #7 | (#1) and (#2 or #3 or #4 or #5) and #6 | 1149 |

**Table S3. Participant characteristics and treatment background of included studies**

| Study ID | Diabetes status | CKD status | Follow-up duration | Background therapy |
| --- | --- | --- | --- | --- |
| Abraham 2021 | 40.60% | NR | 12 weeks | ACEi, ARB, ARNi, beta-blockers, MRA, loop or high-ceiling diuretics, thiazide or low-ceiling diuretics, lipid-lowering agents |
| [McMurray 2024](https://pubmed.ncbi.nlm.nih.gov/?sort=fauth&size=20&term=McMurray+JJV&cauthor_id=38059368) | 44.20% | NR | 16 weeks | ACEi, ARB, ARNi, MRA, loop diuretics, beta-blockers, ICD, CRT |
| [Nassif 2021](https://pubmed.ncbi.nlm.nih.gov/?sort=fauth&size=20&term=Nassif+ME&cauthor_id=34711976) | 55.90% | NR | 12 weeks | ACEi/ARB, ARNi, beta-blockers, hydralazine, long-acting nitrates, MRA, loop diuretics, lipid-lowering agents, anticoagulants |
| [Ovchinnikov 2025](https://pubmed.ncbi.nlm.nih.gov/?sort=fauth&size=20&term=Ovchinnikov+A&cauthor_id=40346546) | 100% | 31.40% | 26 weeks | ACEi/ARB, ARNi, beta-blockers, loop diuretics, thiazide or thiazide-like diuretics, spironolactone, statins, calcium channel blockers, metformin, insulin, sulfonylureas, DPP-4i, GLP-1RAs |
| Solomon 2022 | 44.80% | NR | 120 weeks | NR |
| Solomon 2024 | 40.60% | NR | 138 weeks | beta-blockers, ACEi, ARB, ARNi, calcium channel blockers, SGLT2i, loop diuretics, thiazide diuretics, potassium supplements, GLP-1RAs |
| Spertus 2022 | 72.10% | NR | 12 weeks | NR |
| Tromp 2024 | 49.10% | 48.10% | 13 weeks | ACEi, ARB, ARNi, loop diuretics, thiazide diuretics, beta-blockers, MRA |
| [Vaduganathan 2025](https://pubmed.ncbi.nlm.nih.gov/?sort=fauth&size=20&term=Vaduganathan+M&cauthor_id=39340828) | 35.20% | 47.01% | 120 weeks | ACEi, ARB, ARNi, beta-blockers, calcium channel blockers, loop diuretics |
| [Chimura 2025](https://pubmed.ncbi.nlm.nih.gov/?sort=fauth&size=20&term=Chimura+M&cauthor_id=40377177) | 40.80% | NR | 120 weeks | ACEi or ARB, beta-blockers, ARNi, SGLT2i, loop diuretics, digoxin, anticoagulants, antiplatelet agents, oral iron |
| [Matsumoto 2025](https://pubmed.ncbi.nlm.nih.gov/?term=) | 40.60% | 48.02% | 120 weeks | ACEi, ACEi or ARB, ARNi, beta-blockers, SGLT2i, loop diuretics, thiazide diuretics, digoxin, amiodarone, sotalol, calcium channel blockers, verapamil, diltiazem, flecainide, anticoagulants, antiplatelet agents, pacemaker |
| Yang 2025 | 40.60% | 48.10% | 52 weeks | diuretics, loop diuretics, digoxin, beta-blockers, ACEi, ARB, ARNi, calcium channel blockers, SGLT2i, pacemaker,CRT-P or CRT-D, ICD |
| Anker 2021 | 49.10% | NR | 159 weeks | NR |
| [Voors 2022](https://pubmed.ncbi.nlm.nih.gov/?term=) | 45.30% | 9.60% | 34 weeks | ACEi and/or ARB and/or ARNi, ACEi, ARB, ARNi, MRA, beta-blockers, loop diuretics |
| Szarek 2021 | NR | NR | 39 weeks | ACEi and/or ARB and/or ARNi, ACEi, ARB, ARNi, MRA, beta-blockers, loop diuretics |
| Bhatt 2020 | 2.50% | NR | 34 weeks | metformin, sulfonylureas, DPP-4i, insulin, GLP-1RAs, beta-blockers, loop diuretics, other diuretics |
| [Yang 2022](https://pubmed.ncbi.nlm.nih.gov/?sort=fauth&size=50&term=Yang+M&cauthor_id=36342375) | 39.90% | 52.20% | 34 weeks | ACEi, ARB, ARNi, calcium channel blockers, MRA, diuretics, digoxin, beta-blockers, pacemaker, CRT-P or CRT-D, ICD |
| [Singh 2020](https://pubmed.ncbi.nlm.nih.gov/?term=) | 6.26% | NR | 52 weeks | loop diuretics, ACEi/ARB, beta-blockers, MRA, metformin, other oral hypoglycemic agents, insulin |
| Massie 2008 | 27.40% | 30.20% | 146 weeks | diuretics, spironolactone, ACEi, digoxin, beta-blockers, antiarrhythmic agents, calcium channel blockers, nitrates, oral anticoagulants, antiplatelet agents, lipid-lowering agents |
| Cleland 2006 | 20.60% | NR | 52 weeks | aspirin, oral anticoagulants, beta-blockers, nitrates, calcium channel blockers, lipid-lowering agents, oral hypoglycemic agents, insulin, loop diuretics, thiazide diuretics, low-dose spironolactone, digoxin |
| Yusuf 2003 | 28.30% | NR | 69 weeks | ACEi, beta-blockers, diuretics, spironolactone, digoxin/digitalis glycosides, calcium channel blockers, other vasodilators, oral anticoagulants, antiarrhythmic agents, aspirin, other antiplatelet agents, lipid-lowering agents |
| Parthasarathy 2009 | 18.03% | NR | 14 weeks | ACEi, beta-blockers, ACEi and/or beta-blockers |
| Zi 2003 | 14.90% | NR | 26 weeks | beta-blockers, calcium channel blockers, diuretics, digoxin |
| Ledwidge 2023 | 24.02% | 18.80% | 77 weeks | beta-blockers, calcium channel blockers, statins, thiazide diuretics, aspirin, non-aspirin antiplatelet agents, DOACs, warfarin, oral antidiabetic agents, insulin |
| [Mentz 2023](https://pubmed.ncbi.nlm.nih.gov/?sort=fauth&size=20&term=Mentz+RJ&cauthor_id=37212758) | 48.50% | NR | 16 weeks | ACEi or ARB, MRA, beta-blockers, SGLT2i, loop diuretics |
| Solomon 2020 | 43.03% | NR | 34 weeks | ACEi, ARB, MRA, beta-blockers |
| Solomon 2012 | 37.80% | 41.50% | 36 weeks | ACEi, ARB, MRA, beta-blockers, diuretics |

ACEi: angiotensin-converting enzyme inhibitors; ARB: angiotensin receptor blockers; ARNi: angiotensin receptor–neprilysin inhibitors; MRA: mineralocorticoid receptor antagonists; SGLT2i: sodium–glucose cotransporter 2 inhibitors; DPP-4i: dipeptidyl peptidase-4 inhibitors; GLP-1RAs: glucagon-like peptide-1 receptor agonists; DOACs: Direct Oral Anticoagulants; ICD: implantable cardioverter-defibrillator; CRT: cardiac resynchronization therapy; CRT-P: cardiac resynchronization therapy pacemaker; CRT-D: cardiac resynchronization therapy defibrillator; NR: not reported.

**Table S4. League table for cardiovascular death**

| Placebo | 0.89 (0.82,0.95) | 0.88 (0.77,1.00) | 0.86 (0.72,1.03) | 0.85 (0.66,1.11) | 0.50 (0.09,2.76) | 0.99 (0.86,1.14) | 0.90 (0.71,1.14) |
| --- | --- | --- | --- | --- | --- | --- | --- |
| 1.13 (1.05,1.22) | Finerenone | 0.99 (0.85,1.15) | 0.97 (0.80,1.18) | 0.96 (0.73,1.27) | 0.56 (0.10,3.12) | 1.12 (0.96,1.31) | 1.02 (0.79,1.31) |
| 1.14 (1.00,1.30) | 1.01 (0.87,1.17) | Dapagliflozin | 0.98 (0.78,1.22) | 0.97 (0.72,1.30) | 0.57 (0.10,3.15) | 1.13 (0.93,1.37) | 1.03 (0.78,1.35) |
| 1.16 (0.97,1.39) | 1.03 (0.85,1.25) | 1.02 (0.82,1.27) | Empagliflozin | 0.99 (0.72,1.36) | 0.58 (0.10,3.23) | 1.15 (0.92,1.45) | 1.05 (0.78,1.41) |
| 1.17 (0.90,1.53) | 1.04 (0.79,1.37) | 1.03 (0.77,1.38) | 1.01 (0.73,1.39) | Sotagliflozin | 0.59 (0.10,3.30) | 1.16 (0.86,1.57) | 1.06 (0.74,1.51) |
| 2.00 (0.36,11.03) | 1.77 (0.32,9.78) | 1.76 (0.32,9.75) | 1.72 (0.31,9.58) | 1.71 (0.30,9.60) | Canagliflozin | 1.99 (0.36,11.01) | 1.80 (0.32,10.12) |
| 1.01 (0.88,1.16) | 0.89 (0.76,1.04) | 0.89 (0.73,1.07) | 0.87 (0.69,1.09) | 0.86 (0.64,1.16) | 0.50 (0.09,2.79) | RASi | 0.91 (0.75,1.10) |
| 1.11 (0.87,1.41) | 0.98 (0.76,1.26) | 0.97 (0.74,1.28) | 0.95 (0.71,1.29) | 0.95 (0.66,1.35) | 0.55 (0.10,3.11) | 1.10 (0.91,1.34) | ARNI |

**Table S5. League table for worsening heart failure events**

| Placebo | 0.75 (0.71,0.79) | 0.71 (0.65,0.79) | 0.73 (0.50,1.08) | 0.64 (0.48,0.84) | 1.58 (0.85,2.97) | 0.90 (0.78,1.05) | 0.78 (0.62,0.99) |
| --- | --- | --- | --- | --- | --- | --- | --- |
| 1.34 (1.27,1.41) | Finerenone | 0.95 (0.85,1.07) | 0.98 (0.66,1.46) | 0.85 (0.64,1.12) | 2.12 (1.13,3.98) | 1.21 (1.03,1.42) | 1.05 (0.82,1.33) |
| 1.40 (1.27,1.55) | 1.05 (0.94,1.17) | Dapagliflozin | 1.03 (0.69,1.54) | 0.89 (0.67,1.19) | 2.22 (1.18,4.20) | 1.27 (1.06,1.52) | 1.10 (0.85,1.41) |
| 1.36 (0.92,2.02) | 1.02 (0.69,1.51) | 0.97 (0.65,1.46) | Empagliflozin | 0.87 (0.54,1.40) | 2.16 (1.03,4.53) | 1.23 (0.81,1.88) | 1.07 (0.67,1.68) |
| 1.57 (1.20,2.07) | 1.18 (0.89,1.55) | 1.12 (0.84,1.50) | 1.15 (0.71,1.86) | Sotagliflozin | 2.49 (1.26,4.95) | 1.42 (1.04,1.95) | 1.23 (0.86,1.76) |
| 0.63 (0.34,1.18) | 0.47 (0.25,0.89) | 0.45 (0.24,0.85) | 0.46 (0.22,0.97) | 0.40 (0.20,0.80) | Canagliflozin | 0.57 (0.30,1.09) | 0.49 (0.25,0.96) |
| 1.11 (0.95,1.29) | 0.83 (0.70,0.97) | 0.79 (0.66,0.94) | 0.81 (0.53,1.23) | 0.70 (0.51,0.96) | 1.75 (0.92,3.34) | RASi | 0.86 (0.72,1.03) |
| 1.28 (1.01,1.62) | 0.96 (0.75,1.21) | 0.91 (0.71,1.18) | 0.94 (0.59,1.48) | 0.81 (0.57,1.17) | 2.03 (1.04,3.96) | 1.16 (0.97,1.38) | ARNI |

**Table S6. League table for the composite renal outcome**

| Placebo | 1.42 (1.10,1.84) | 0.94 (0.77,1.16) | 0.88 (0.70,1.11) | 0.93 (0.53,1.63) | 1.21 (0.85,1.73) | 0.69 (0.43,1.11) |
| --- | --- | --- | --- | --- | --- | --- |
| 0.70 (0.54,0.91) | Finerenone | 0.66 (0.48,0.92) | 0.62 (0.44,0.88) | 0.66 (0.36,1.21) | 0.85 (0.55,1.33) | 0.48 (0.28,0.83) |
| 1.06 (0.87,1.30) | 1.51 (1.08,2.09) | Dapagliflozin | 0.94 (0.69,1.27) | 0.99 (0.55,1.79) | 1.29 (0.85,1.94) | 0.73 (0.44,1.22) |
| 1.13 (0.90,1.43) | 1.61 (1.14,2.27) | 1.07 (0.78,1.45) | Empagliflozin | 1.06 (0.58,1.93) | 1.37 (0.90,2.10) | 0.78 (0.46,1.32) |
| 1.07 (0.62,1.87) | 1.52 (0.83,2.81) | 1.01 (0.56,1.83) | 0.95 (0.52,1.73) | Sotagliflozin | 1.30 (0.67,2.52) | 0.74 (0.36,1.54) |
| 0.82 (0.58,1.18) | 1.17 (0.75,1.82) | 0.78 (0.52,1.17) | 0.73 (0.48,1.11) | 0.77 (0.40,1.49) | RASi | 0.57 (0.41,0.78) |
| 1.45 (0.90,2.34) | 2.06 (1.20,3.55) | 1.37 (0.82,2.30) | 1.28 (0.76,2.18) | 1.35 (0.65,2.81) | 1.76 (1.29,2.42) | ARNI |

**Table S7. League table for all-cause mortality**

| Placebo | 0.93 (0.87,0.99) | 0.94 (0.85,1.03) | 0.93 (0.81,1.07) | 0.85 (0.60,1.20) | 1.02 (0.88,1.19) | 0.99 (0.79,1.23) |
| --- | --- | --- | --- | --- | --- | --- |
| 1.08 (1.01,1.15) | Finerenone | 1.01 (0.90,1.13) | 1.00 (0.86,1.17) | 0.91 (0.64,1.31) | 1.10 (0.94,1.29) | 1.07 (0.85,1.34) |
| 1.07 (0.97,1.17) | 0.99 (0.88,1.11) | Dapagliflozin | 0.99 (0.84,1.17) | 0.90 (0.63,1.30) | 1.09 (0.91,1.30) | 1.06 (0.83,1.34) |
| 1.08 (0.94,1.24) | 1.00 (0.86,1.16) | 1.01 (0.85,1.20) | Empagliflozin | 0.91 (0.62,1.33) | 1.10 (0.90,1.35) | 1.07 (0.82,1.38) |
| 1.18 (0.83,1.68) | 1.10 (0.77,1.57) | 1.11 (0.77,1.59) | 1.10 (0.75,1.60) | Sotagliflozin | 1.21 (0.82,1.77) | 1.17 (0.77,1.77) |
| 0.98 (0.84,1.14) | 0.91 (0.77,1.07) | 0.92 (0.77,1.09) | 0.91 (0.74,1.11) | 0.83 (0.57,1.21) | RASi | 0.97 (0.82,1.14) |
| 1.01 (0.81,1.26) | 0.94 (0.75,1.18) | 0.95 (0.75,1.20) | 0.94 (0.72,1.22) | 0.86 (0.57,1.30) | 1.03 (0.88,1.21) | ARNI |

**Table S8. League table for total heart failure hospitalizations**

| Placebo | 0.87 (0.78,0.97) | 0.76 (0.68,0.85) | 0.72 (0.63,0.83) | 0.53 (0.45,0.63) | 0.80 (0.66,0.97) | 0.95 (0.82,1.09) | 0.76 (0.64,0.91) |
| --- | --- | --- | --- | --- | --- | --- | --- |
| 1.15 (1.03,1.27) | Finerenone | 0.87 (0.75,1.01) | 0.83 (0.69,0.99) | 0.61 (0.50,0.74) | 0.91 (0.74,1.14) | 1.08 (0.91,1.29) | 0.87 (0.71,1.08) |
| 1.32 (1.18,1.47) | 1.15 (0.99,1.34) | Dapagliflozin | 0.95 (0.80,1.13) | 0.70 (0.57,0.85) | 1.05 (0.84,1.31) | 1.24 (1.04,1.48) | 1.00 (0.81,1.24) |
| 1.38 (1.21,1.59) | 1.21 (1.02,1.44) | 1.05 (0.88,1.25) | Empagliflozin | 0.73 (0.59,0.91) | 1.11 (0.87,1.40) | 1.31 (1.08,1.59) | 1.05 (0.84,1.32) |
| 1.89 (1.60,2.23) | 1.65 (1.35,2.01) | 1.44 (1.17,1.76) | 1.36 (1.10,1.70) | Sotagliflozin | 1.51 (1.17,1.95) | 1.79 (1.44,2.22) | 1.44 (1.12,1.84) |
| 1.25 (1.03,1.51) | 1.09 (0.88,1.36) | 0.95 (0.76,1.18) | 0.90 (0.72,1.14) | 0.66 (0.51,0.85) | Canagliflozin | 1.18 (0.94,1.50) | 0.95 (0.73,1.24) |
| 1.06 (0.92,1.21) | 0.92 (0.78,1.10) | 0.80 (0.67,0.96) | 0.76 (0.63,0.93) | 0.56 (0.45,0.70) | 0.84 (0.67,1.07) | RASi | 0.81 (0.72,0.91) |
| 1.31 (1.09,1.57) | 1.15 (0.93,1.42) | 1.00 (0.81,1.23) | 0.95 (0.76,1.19) | 0.70 (0.54,0.89) | 1.05 (0.81,1.36) | 1.24 (1.10,1.40) | ARNI |

**Table S9. League table for adverse events**

| **Placebo** | 0.92 (0.85,0.99) | 0.94 (0.86,1.04) | 0.72 (0.54,0.96) | 0.91 (0.72,1.15) | 1.41 (1.16,1.70) | 1.20 (1.02,1.42) | 1.01 (0.80,1.29) |
| --- | --- | --- | --- | --- | --- | --- | --- |
| 1.09 (1.01,1.18) | **Finerenone** | 1.03 (0.91,1.16) | 0.79 (0.58,1.06) | 0.99 (0.78,1.27) | 1.53 (1.25,1.88) | 1.31 (1.10,1.57) | 1.11 (0.86,1.42) |
| 1.06 (0.96,1.17) | 0.97 (0.86,1.10) | **Dapagliflozin** | 0.76 (0.56,1.04) | 0.97 (0.75,1.24) | 1.49 (1.21,1.85) | 1.28 (1.06,1.54) | 1.08 (0.83,1.39) |
| 1.39 (1.04,1.86) | 1.27 (0.94,1.72) | 1.31 (0.96,1.78) | **Empagliflozin** | 1.27 (0.87,1.83) | 1.95 (1.38,2.76) | 1.67 (1.20,2.33) | 1.41 (0.97,2.05) |
| 1.10 (0.87,1.38) | 1.01 (0.79,1.28) | 1.03 (0.81,1.33) | 0.79 (0.55,1.14) | **Sotagliflozin** | 1.54 (1.15,2.08) | 1.32 (1.00,1.75) | 1.11 (0.80,1.55) |
| 0.71 (0.59,0.86) | 0.65 (0.53,0.80) | 0.67 (0.54,0.83) | 0.51 (0.36,0.72) | 0.65 (0.48,0.87) | **Canagliflozin** | 0.86 (0.67,1.10) | 0.72 (0.53,0.98) |
| 0.83 (0.71,0.98) | 0.76 (0.64,0.91) | 0.78 (0.65,0.95) | 0.60 (0.43,0.83) | 0.76 (0.57,1.00) | 1.17 (0.91,1.50) | **RAsi** | 0.84 (0.71,1.00) |
| 0.99 (0.78,1.25) | 0.90 (0.70,1.16) | 0.93 (0.72,1.20) | 0.71 (0.49,1.03) | 0.90 (0.64,1.25) | 1.39 (1.02,1.88) | 1.19 (1.00,1.41) | **ARNI** |

**Figure S1. Forest plots**


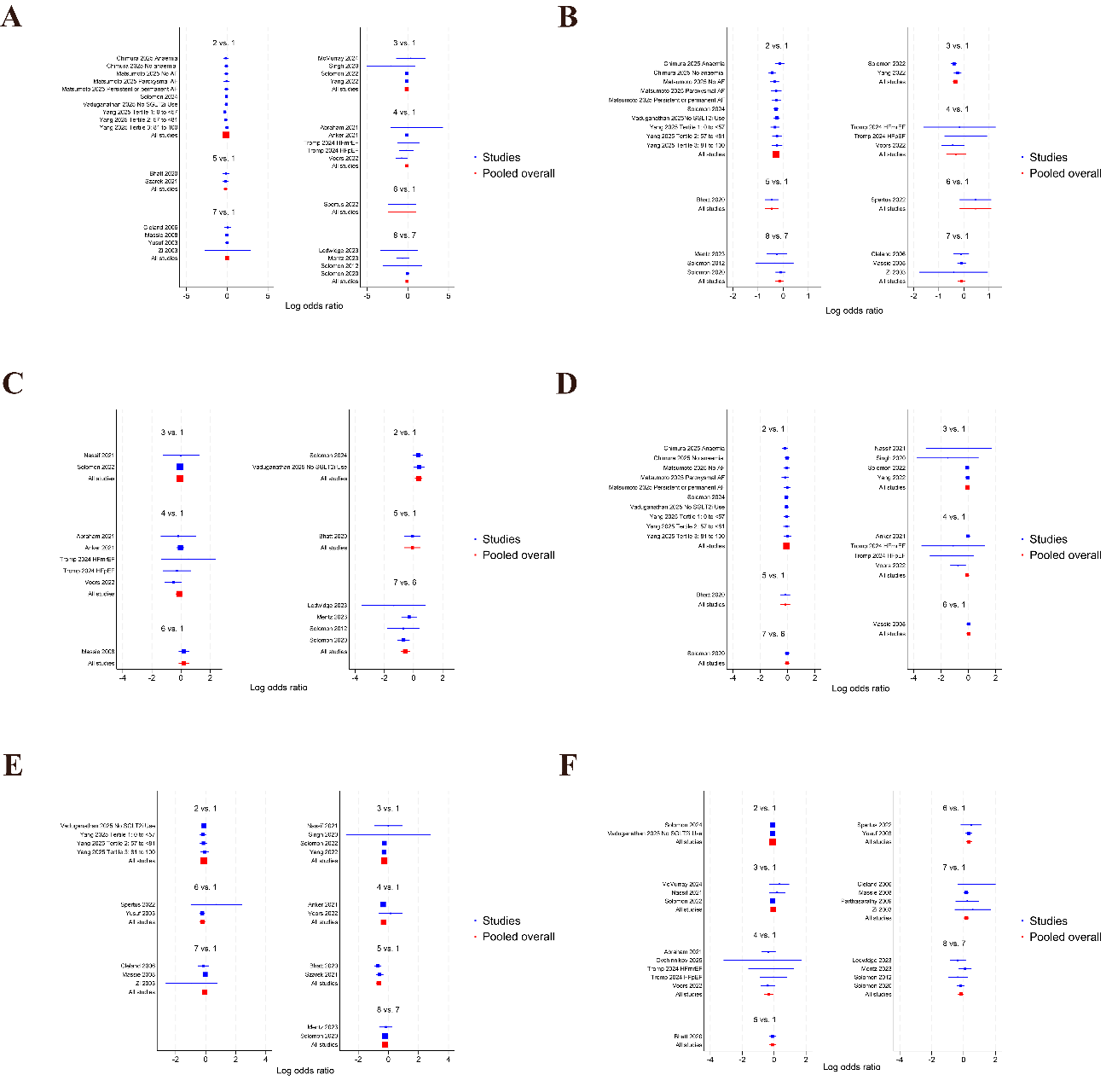


A: Cardiovascular death; B: Worsening heart failure events; C: Composite renal outcome; D: All-cause mortality; E: Total heart failure hospitalizations; F: Adverse events.

**Figure S2. SUCRA ranking plot**


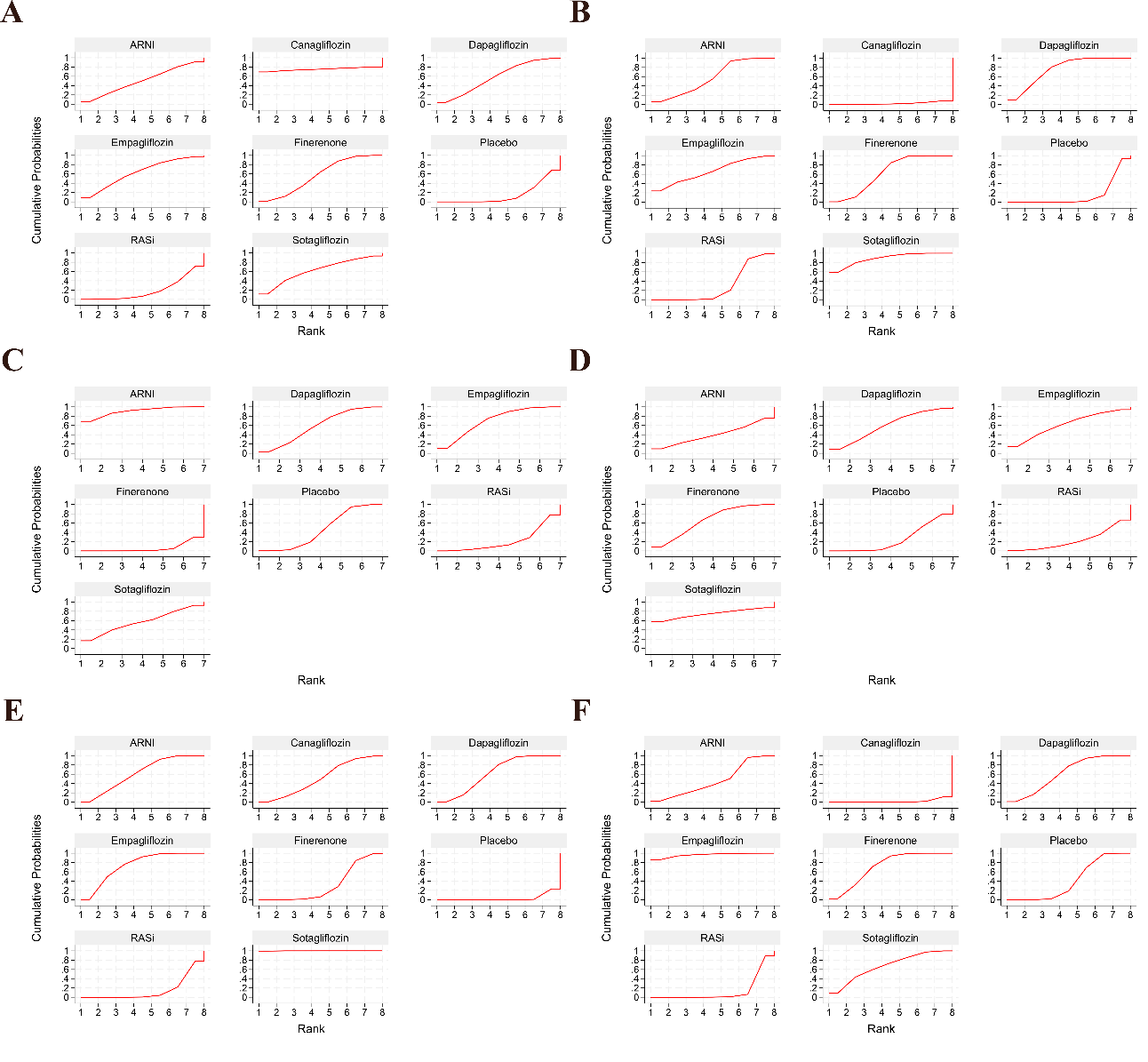


A: Cardiovascular death; B: Worsening heart failure events; C: Composite renal outcome; D: All-cause mortality; E: Total heart failure hospitalizations; F: Adverse events.

**Figure S3. Network plot for sensitivity analysis**

**
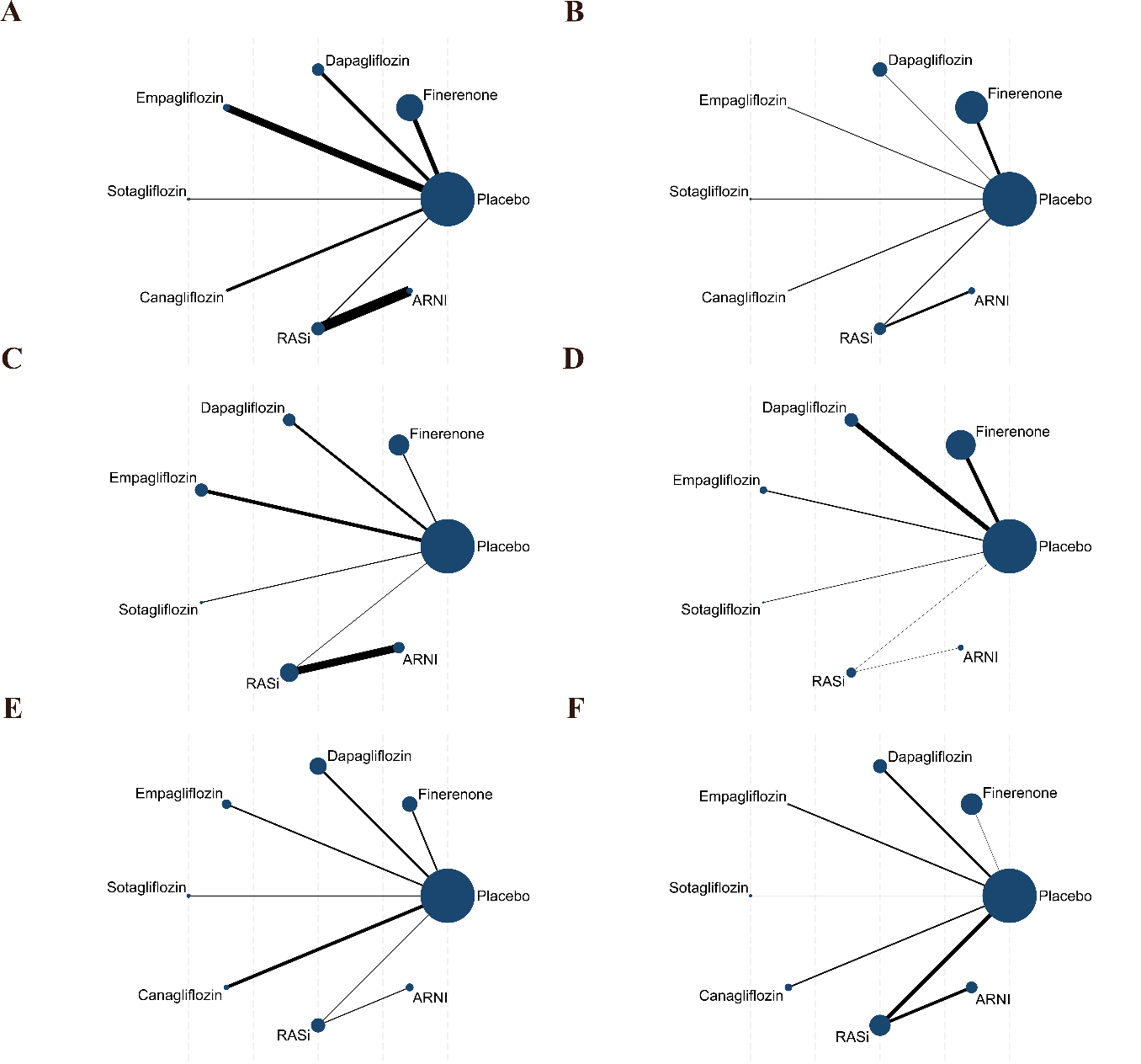
**

A: Cardiovascular death; B: Worsening heart failure events; C: Composite renal outcome; D: All-cause mortality; E: Total heart failure hospitalizations; F: Adverse events.

**Figure S4. Pairwise comparison forest plot for sensitivity analysis**

**
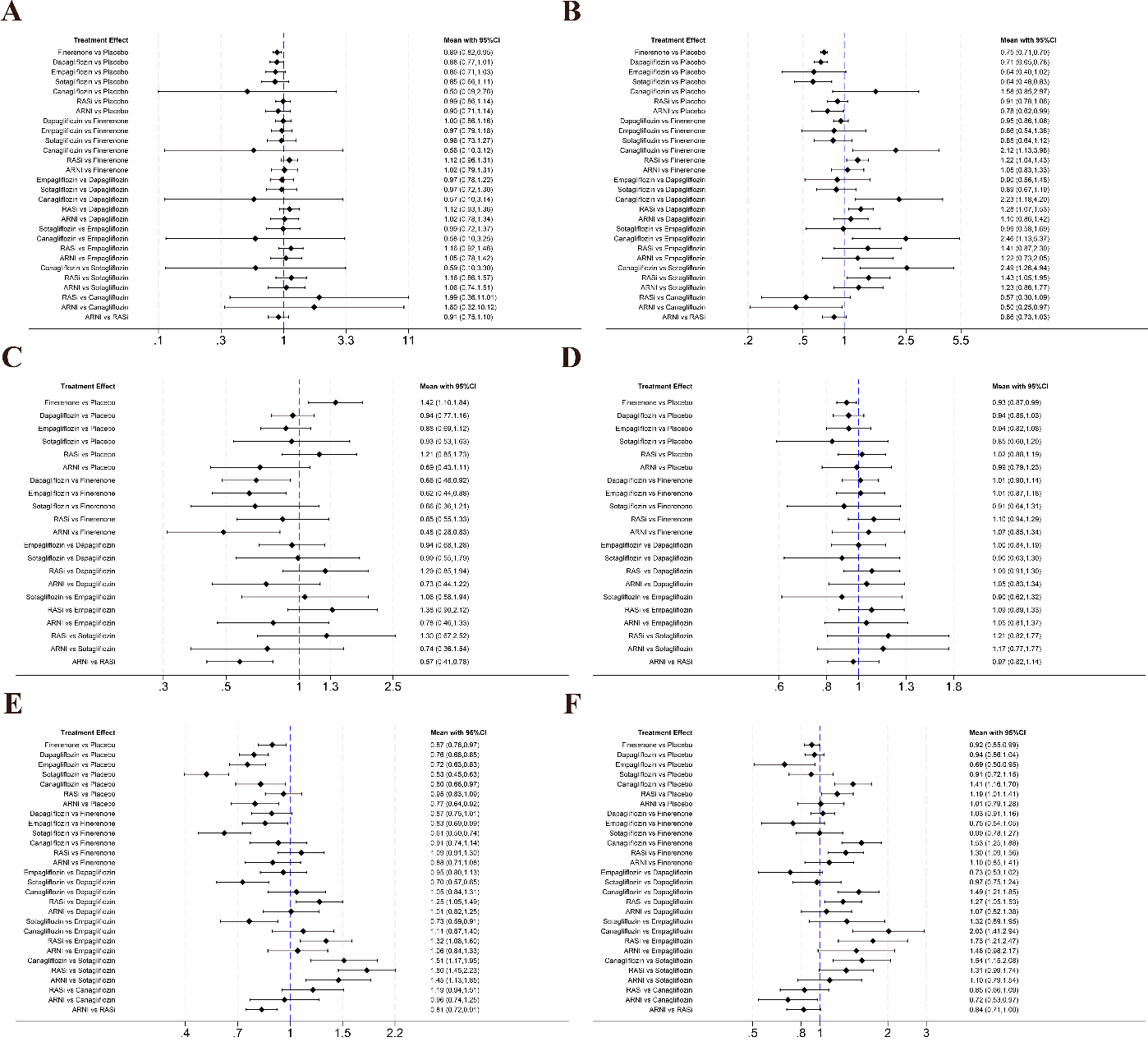
**

A: Cardiovascular death; B: Worsening heart failure events; C: Composite renal outcome; D: All-cause mortality; E: Total heart failure hospitalizations; F: Adverse events.

**Figure S5. Funnel plot for sensitivity analysis**


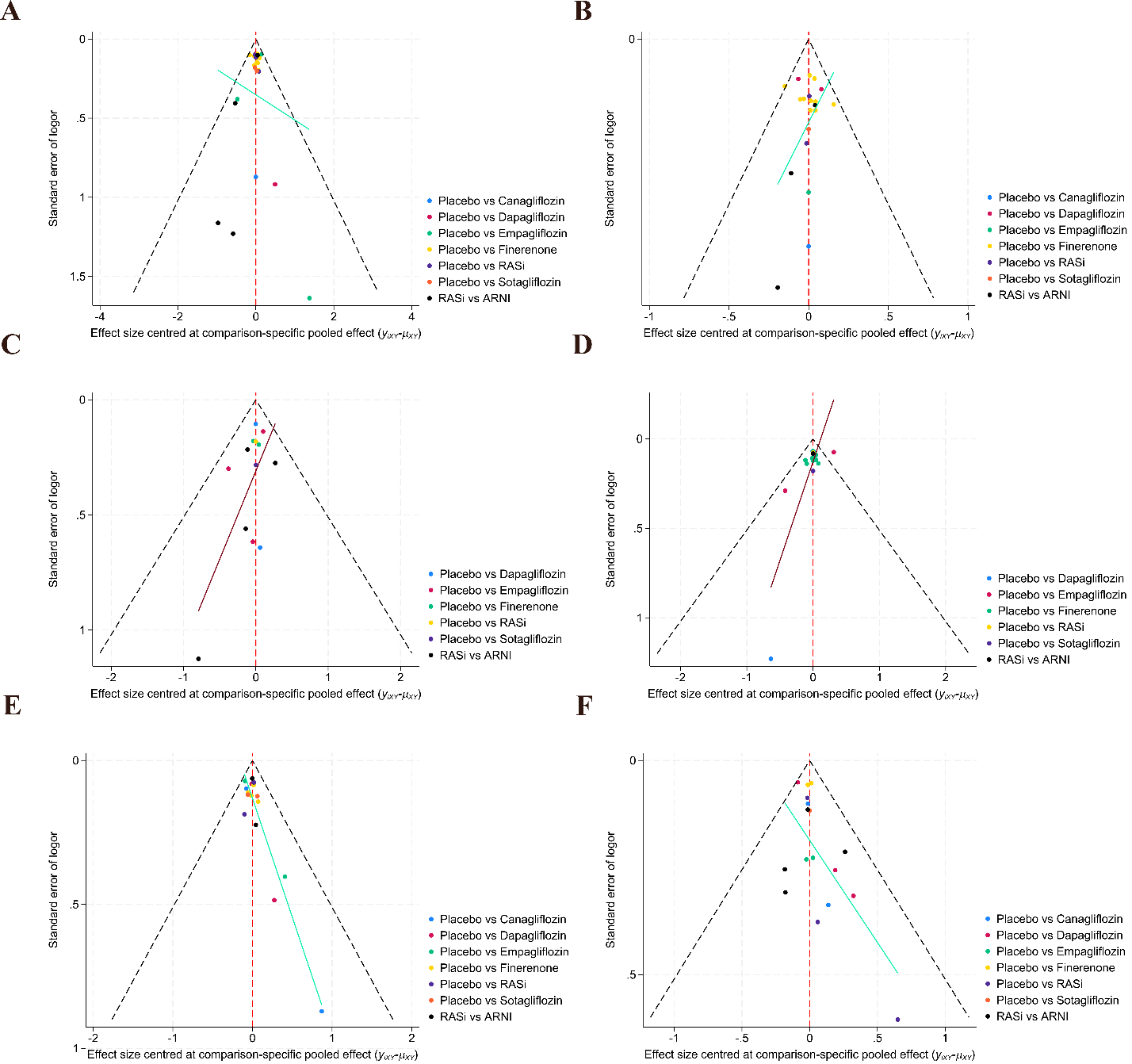


A: Cardiovascular death; B: Worsening heart failure events; C: Composite renal outcome; D: All-cause mortality; E: Total heart failure hospitalizations; F: Adverse events.
